# Supplementary material for: CE-BLAST makes it possible to compute antigenic similarity for newly emerging pathogens
Source: Nat Commun. 2018 May 2;9:1772. doi: 10.1038/s41467-018-04171-2 (PMC5932059; doi:10.1038/s41467-018-04171-2)
Supplement: Supplementary file 1 — Supplementary Information [file 41467_2018_4171_MOESM1_ESM.pdf]

Qiu et al. CE-BLAST makes it possible to compute antigenic similarity for newly emerging pathogens

Supplementary Figures

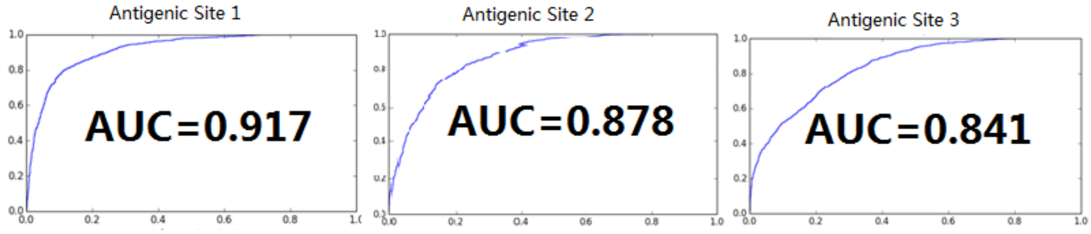

Supplementary Figure 1. Performance of CE-BLAST on different antigenic sites. Site set 1 were based on 16 overlapped sites between Liao et al [1] and Smith et al [2]. Site set 2 were based on 44 sites of Smith et al [2]. Site set 3 were based on 26 sites of Wiley et al [3].

```
                      1        10        20        30        40        50        60
CON_H3                QKLPGNDNSTATLCLGHHAVPNGTIVKTITNDQIEVTNATELVQSSSTGEICDSPHQILD
A/Brisbane/10/2007    QKLPGNDNSTATLCLGHHAVPNGTIVKTITNDQIEVTNATELVQSSSTGEICDSPHQILD
A/Wisconsin/67/2005   QKLPGNDNSTATLCLGHHAVPNGTIVKTITNDQIEVTNATELVQSSSTGGICDSPHQILD
A/Fujian/411/2002     QKLPGNDNSTATLCLGHHAVPNGTIVKTITNDQIEVTNATELVQSSSTGGICDSPHQILD
A/Hong_Kong/1/1968    QDLPGNDNSTATLCLGHHAVPNGTLVKTITDDQIEVTNATELVQSSSTGKICNNPHRILD
A/Philippines/2/1982  QNLPGNDNSTATLCLGHHAVPNGTLVKTITNDQIEVTNATELVQSSSTGRICDSPHRILD
A/Moscow/10/1999      QKLPGNDNSTATLCLGHHAVPNGTLVKTITNDQIEVTNATELVQSSSTGRICDSPHQILD
A/Indiana/08/2011     QKLPGSDNSMATLCLGHHAVPNGTLVKTITDDQIEVTNATELVQSSSTGGICNSPHQILD

                      61       70        80        90        100       110       120
CON_H3                GENCTLIDALLGDPQCDGFQNKKWDLFVERSKAYSNCYPYDVPDYASLRSLVASSGTLEF
A/Brisbane/10/2007    GENCTLIDALLGDPQCDGFQNKKWDLFVERSKAYSNCYPYDVPDYASLRSLVASSGTLEF
A/Wisconsin/67/2005   GENCTLIDALLGDPQCDGFQNKKWDLFVERSKAYSNCYPYDVPDYASLRSLVASSGTLEF
A/Fujian/411/2002     GENCTLIDALLGDPQCDGFQNKKWDLFVERSKAYSNCYPYDVPDYASLRSLVASSGTLEF
A/Hong_Kong/1/1968    GIDCTLIDALLGDPHCDVFQNETWDLFVERSKAFSNCYPYDVPDYASLRSLVASSGTLEF
A/Philippines/2/1982  GKNCTLIDALLGDPHCDGFQNEKWDLFVERSKAFSNCYPYDVPDYASLRSLVASSGTLEF
A/Moscow/10/1999      GENCTLIDALLGDPHCDGFQNKEWDLFVERSKAYSNCYPYDVPDYASLRSLVASSGTLEF
A/Indiana/08/2011     GKNCTLIDALLGDPHCDDFQNKEWDLFVERSTAYSNCYPYYVPDYATLRSLVASSGNLEF

                      121      130       140       150       160       170       180
CON_H3                NNESFNWTGVTQNGTSSACIRRSNNSFFSRLNWLTHLKFKYPALNVTMPNNEQFDKLYIW
A/Brisbane/10/2007    NNESFNWTGVTQNGTSSACIRRSNNSFFSRLNWLTHLKFKYPALNVTMPNNEKFDKLYIW
A/Wisconsin/67/2005   NDESFNWTGVTQNGTSSSCKRRSNNSFFSRLNWLTHLKFKYPALNVTMPNNEKFDKLYIW
A/Fujian/411/2002     NNESFNWTGVTQNGTSSACKRRSNNSFFSKLNWLTHLKFKYPALNVTMPNNEKFDKLYIW
A/Hong_Kong/1/1968    ITEGFTWTGVTQNGGSNACKRGPGSGFFSRLNWLTKSGSTYPVLNVTMPNNDNFDKLYIW
A/Philippines/2/1982  INEGFNWTGVTQSGGSYTCKRGSNNSFFSRLNWLYESESKYPVLNVTMPNNGKFDKLYIW
A/Moscow/10/1999      NNESFNWTGVAQNGTSSSCKRRSIKSFFSRLNWLHQLKYRYPALNVTMPNNDKFDKLYIW
A/Indiana/08/2011     TQESFNWTGVAQGGSSYACRRGSVNSFFSRLNWLYNLNYKYPEQNVTMPNNDKFDKLYIW

                      181      190       200       210       220       230       240
CON_H3                GVHHPGTDNDQIFLYAQASGRITVSTKRSQQTVIPNIGSRPRVRNIPSRISIYWTIVKPG
A/Brisbane/10/2007    GVHHPGTDNDQIFPYAQASGRITVSTKRSQQTVIPNIGSRPRVRNIPSRISIYWTIVKPG
A/Wisconsin/67/2005   GVHHPVTDNDQIFLYAQASGRITVSTKRSQQTVIPNIGSRPRIRNIPSRISIYWTIVKPG
A/Fujian/411/2002     GVHHPGTNNDQISLYAQASGRITVSTKRSQQTVIPNIGSRPRVRDIPSRISIYWTIVKPG
A/Hong_Kong/1/1968    GVHHPSTNQEQTSLYVQASGRVTVSTRRSQQTIIPNIGSRPWVRGMSSRISIYWTIVKPG
A/Philippines/2/1982  GIHHPSTDKEQTNLYIRASGRVTVSTKRSQQTVIPNIGSRPWVRGLSSRISIYWTIVKPG
A/Moscow/10/1999      GVHHPSTDSDQTSLYTQASGRVTVSTKRSQQTVIPNIGSRPWVRGISSRISIYWTIVKPG
A/Indiana/08/2011     GVHHPGTDKDQTNLYVQASGRVIVSTKRSQQTVIPNIGSRPWVRGVSSIISIYWTIVKPG

                      241      250       260       270       280       290       300
CON_H3                DILLINSTGNLIAPRGYFKIRSGKSSIMRSDAPIGKCNSECITPNGSIPNDKPFQNVNRI
A/Brisbane/10/2007    DILLINSTGNLIAPRGYFKIRSGKSSIMRSDAPIGKCNSECITPNGSIPNDKPFQNVNRI
A/Wisconsin/67/2005   DILLINSTGNLIAPRGYFKIRSGKSSIMRSDAPIGKCNSECITPNGSIPNDKPFQNVNRI
A/Fujian/411/2002     DILLINSTGNLIAPRGYFKIRSGKSSIMRSDAPIGKCNSECITPNGSIPNDKPFQNVNRI
A/Hong_Kong/1/1968    DVLVINSNGNLIAPRGYFKMRTGKSSIMRSDAPIDTCISECITPNGSIPNDKPFQNVNKI
A/Philippines/2/1982  DILLINSTGNLIAPRGYFKIRTGKSSIMRSDAPIGTCSSECITPNGSIPNDKPFQNVNKI
A/Moscow/10/1999      DILLINSTGNLIAPRGYFKIRSGKSSIMRSDAPIGKCNSECITPNGSIPNDKPFQNVNRI
A/Indiana/08/2011     DILLINSTGNLIAPRGYFKIQSGKSSIMRSDAHIDECNSECITPNGSIPNDKPFQNVNKI

                      301      310       320      329
CON_H3                TYGACPRYVKQNTLKLATGMRNVPEKQTR
A/Brisbane/10/2007    TYGACPRYVKQNTLKLATGMRNVPEKQTR
A/Wisconsin/67/2005   TYGACPRYVKQNTLKLATGMRNVPEKQTR
A/Fujian/411/2002     TYGACPRYVKQNTLKLATGMRNVPEKQTR
A/Hong_Kong/1/1968    TYGACPKYVKQNTLKLATGMRNVPEKQTR
A/Philippines/2/1982  TYGACPRYVKQNTLKLATGMRNVPEKQTR
A/Moscow/10/1999      TYGACPRYVKQNTLKLATGMRNVPEKQTR
A/Indiana/08/2011     TYGACPRYVKQNTLKLATGMRNVPEKQTR
```

Supplementary Figure 2. Sequence comparison of “Con H3” and seven reference strains.

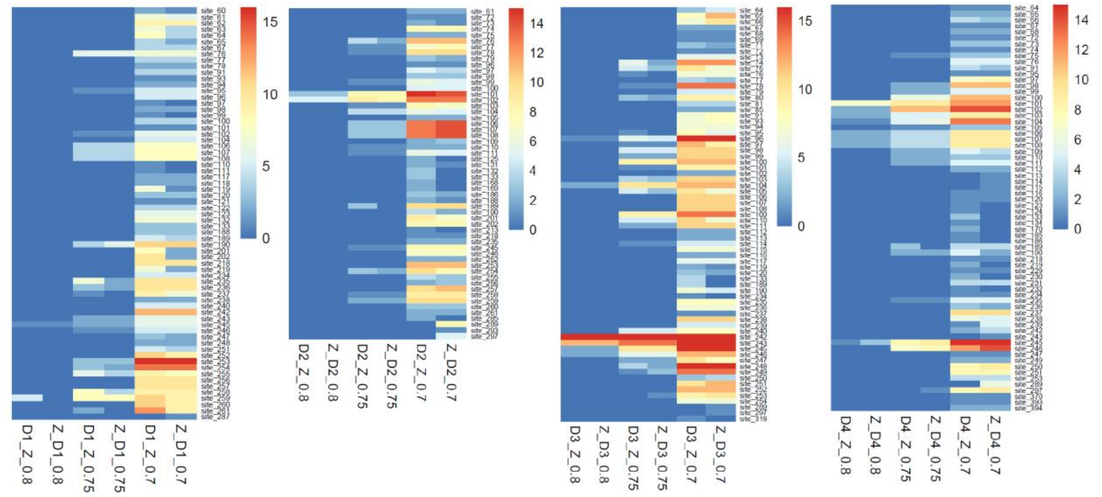

Supplementary Figure 3. Potential cross-reactive epitope detected based on monomer structure of E protein. D1~D4 means Dengue virus serotype 1~4, Z means Zika virus. For each heat map, the horizontal axis labeled epitope areas with different numbers while vertical axis means results under different threshold. Each unit in the heat map shows the number of compared pairs over the certain threshold.

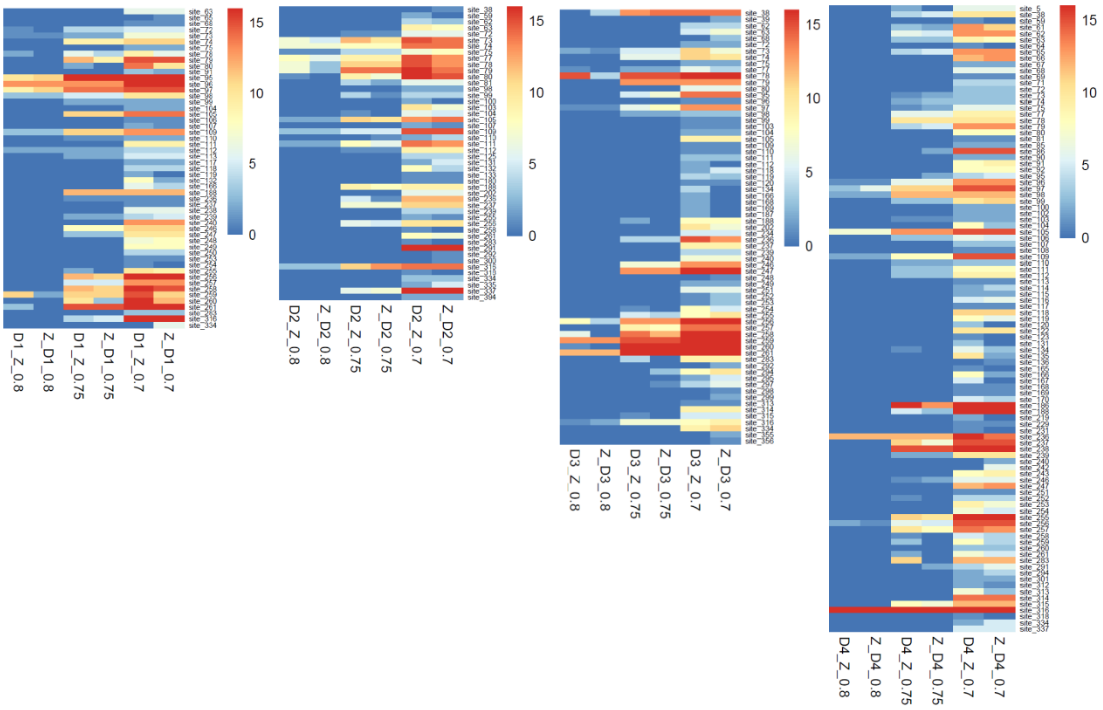

Supplementary Figure 4. Potential cross-reactive epitope detected based on dimer structure of E protein. D1~D4 means Dengue virus serotype 1~4, Z means Zika virus. For each heat map, the horizontal axis labeled epitope areas with different numbers while vertical axis means results under different threshold. Each unit in the heat map shows the number of compared pairs over the certain threshold.

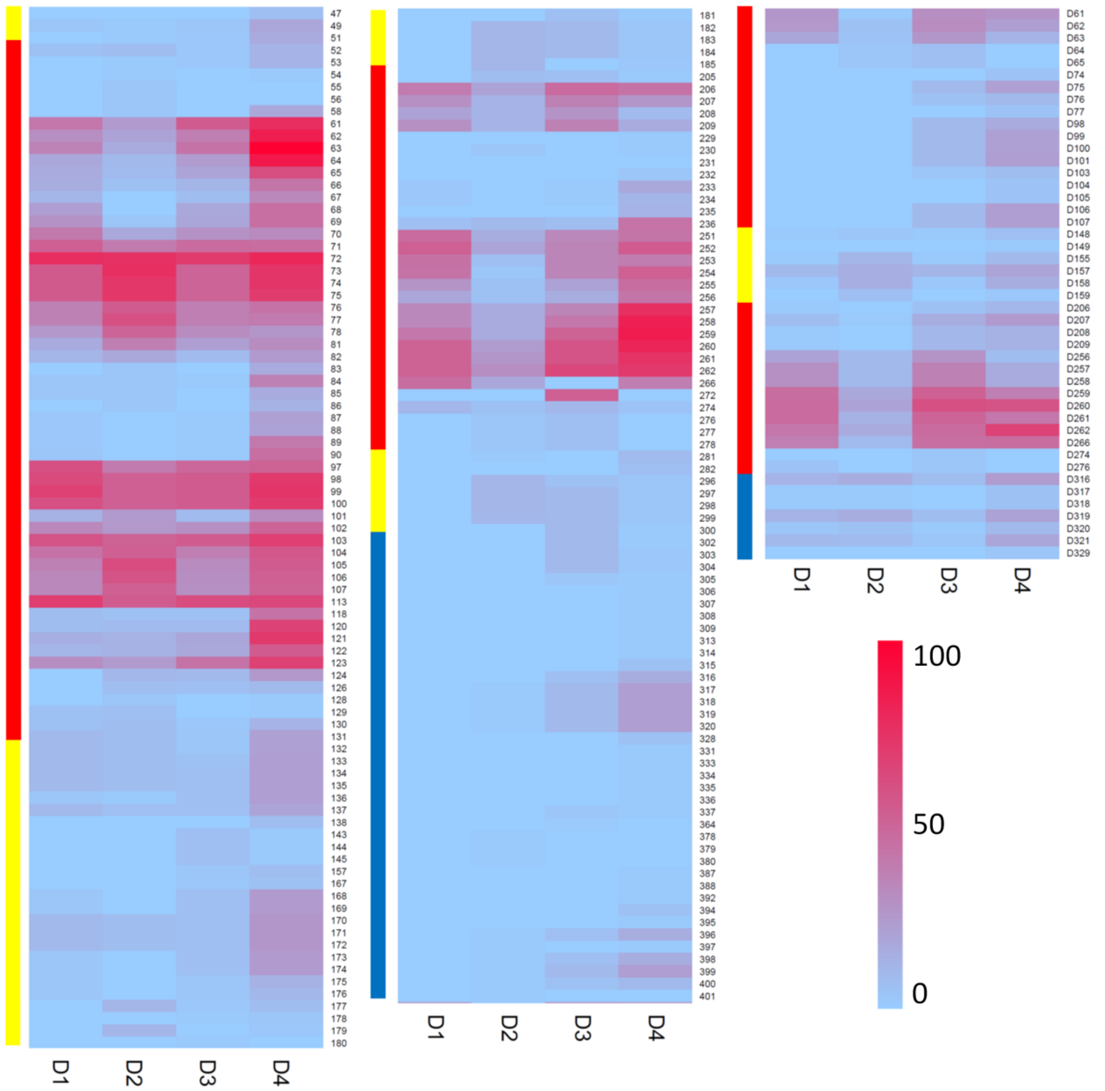

Supplementary Figure 5. Dimer results predicted by CE-BLAST, the horizontal axis represents for four serotypes of DENV while the vertical axis displayed the signal intensity of each surface residue. Residues labeled with D means it located on the corresponding dimer chain. Signal intensity of each residue was normalized and marked in the heat map.

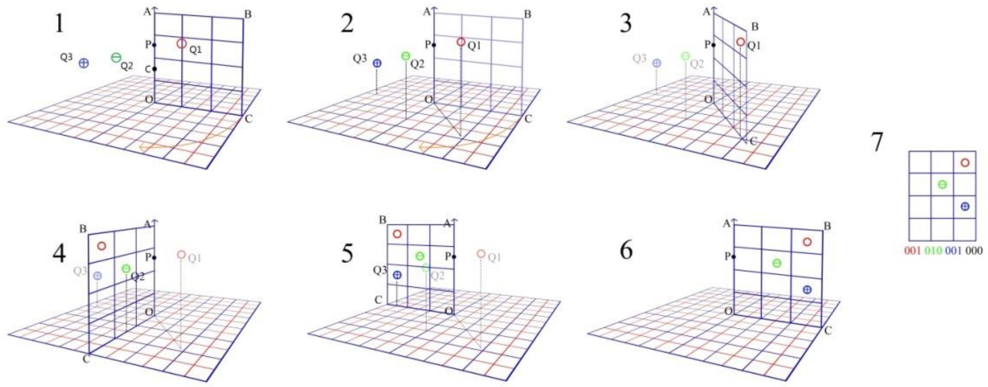

Supplementary Figure 6. Illustration of structural fingerprint generation for target residue P through spin-image strategy. Step1: spin-image plane for target residue P. C is supposed to be the geometric center of the epitope which contains 4 residues: P, Q1,

Q2 and Q3. OP, PA and AB were set as 30 Å, 10Å and 20Å respectively. Step 2-6: Rotating spin-image plane around vector $\overrightarrow{CP}$, all neighboring residue Q1, Q2 and Q3 can be punched into the grid position in the spin-image plane. Step 7: Illustration of "Spin-image" fingerprint generation. The numbers of punched residues were counted for each grid to generate fingerprint.

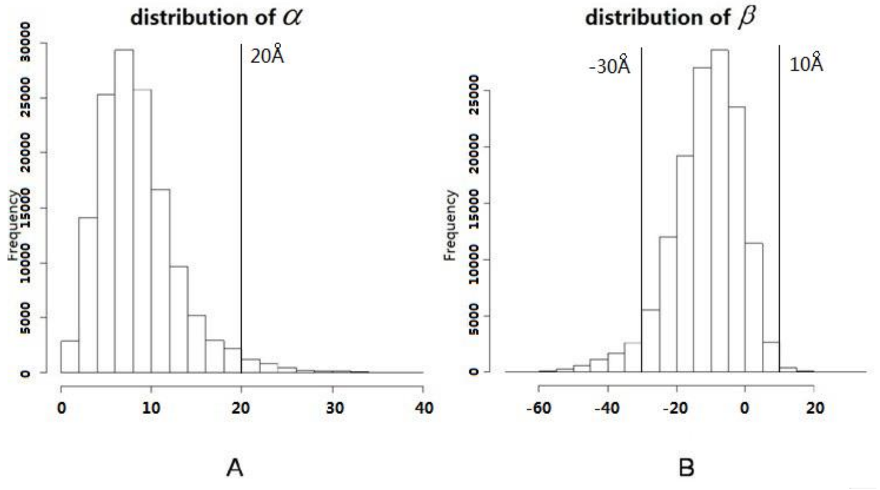

Supplementary Figure 7. Coordinates distribution of epitope amino acid projection: Figure A represents the distribution of horizontal axis α. Figure B represents the distribution of vertical axis β. The range of 20Å as horizontal axis and -30Å to 10Å as vertical axis can contains at least 95% of the epitope residues.

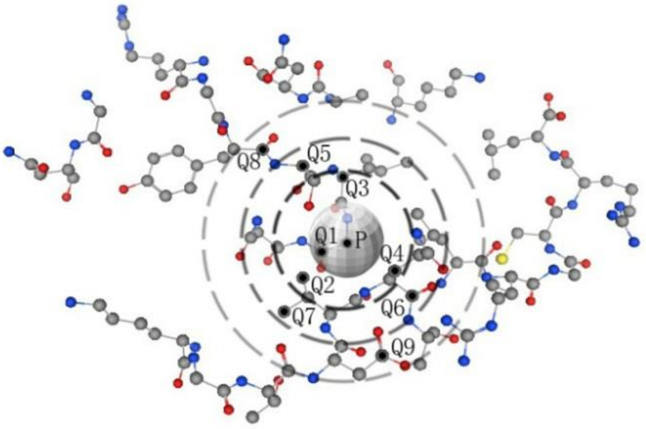

Supplementary Figure 8. Illustration of physical-chemical fingerprint generation. P represents the target residue, while Q1~Q9 in the figure represent the neighboring residues. For example, the hydrophobicity parameter of shell 1 is the accumulation of hydrophobicity index of P and Q1. Amino Acid Indexes of ARGP820101, FAUJ880109 and FAUJ880108 were taken to calculate Physical-chemical properties.

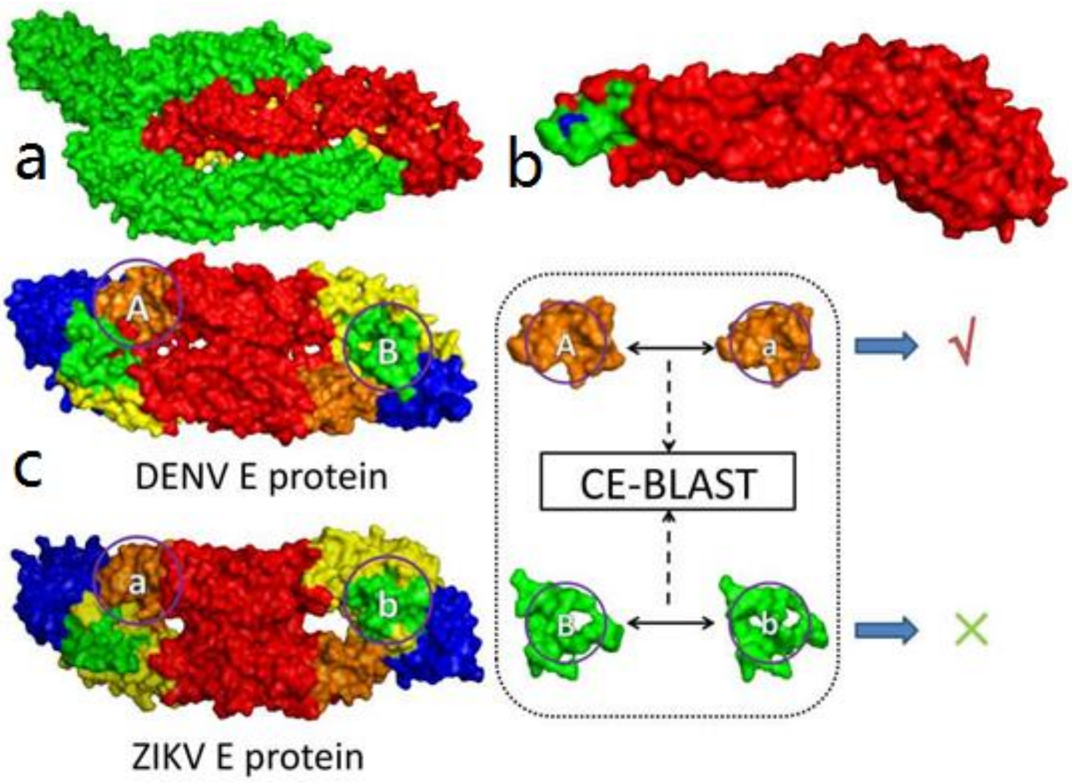

Supplementary Figure 9. Workflow of potential cross-reactive epitope detection. (a) Trimer structure of DENV E protein (PDB: 3j27). Chain C was marked with red (artificially selected surface) and yellow (the rest) colors. (b) Monomer structure of DENV E protein (PDB: 3j27 chain C). Residue selected as center of an epitope area was marked as blue while its neighborhood residues were marked as green. (c) Each comparable epitope areas were calculated through CE-BLAST, epitope pairs achieves CE-BLAST score over the threshold will be defined as potential cross-reactive epitope area.

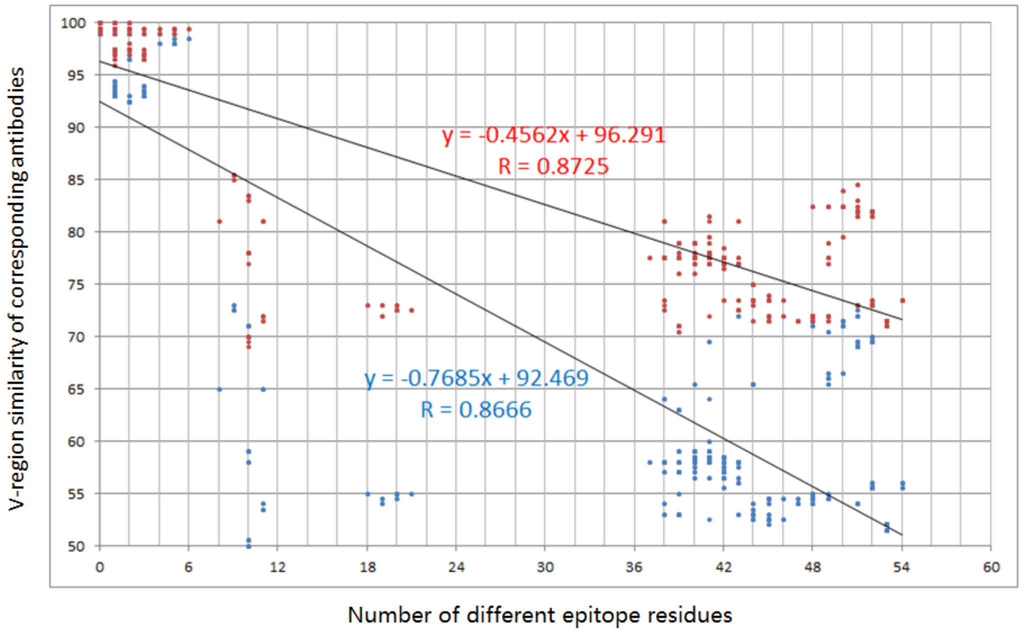

Supplementary Figure 10. Correlations between number of residue difference and V-region similarity of corresponding antibodies. Red scatter diagram represent V-region similarity (positive). Blue scatter diagram represent V-region similarity (identities).

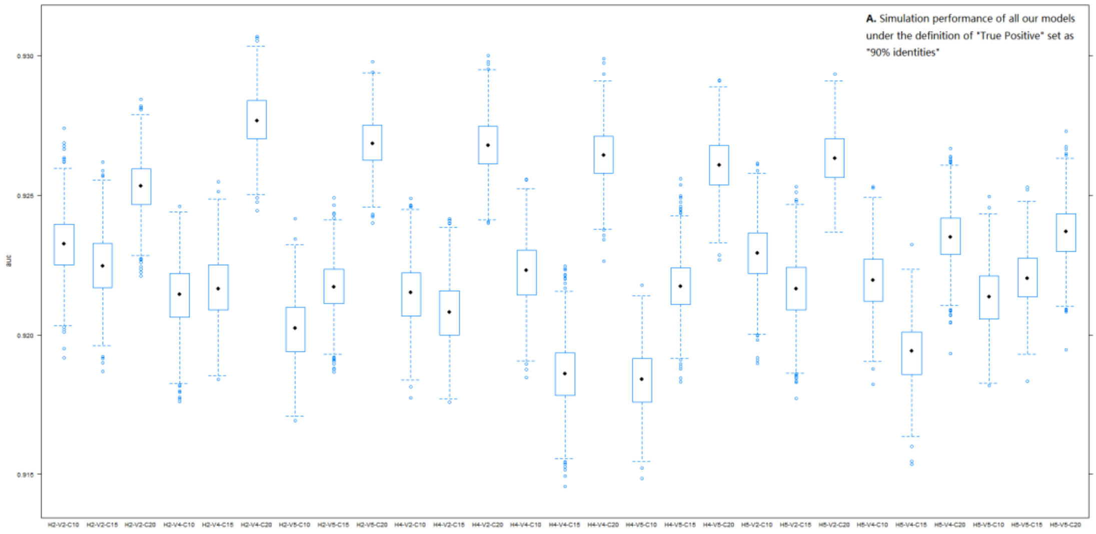
A. Simulation performance of all our models under the definition of "True Positive" set as "90% identities"
auc
H2-V2-C10 H2-V2-C15 H2-V2-C20 H2-V4-C10 H2-V4-C15 H2-V4-C20 H2-V5-C10 H2-V5-C15 H2-V5-C20 H4-V2-C10 H4-V2-C15 H4-V2-C20 H4-V4-C10 H4-V4-C15 H4-V4-C20 H4-V5-C10 H4-V5-C15 H4-V5-C20 H5-V2-C10 H5-V2-C15 H5-V2-C20 H5-V4-C10 H5-V4-C15 H5-V4-C20 H5-V5-C10 H5-V5-C15 H5-V5-C20

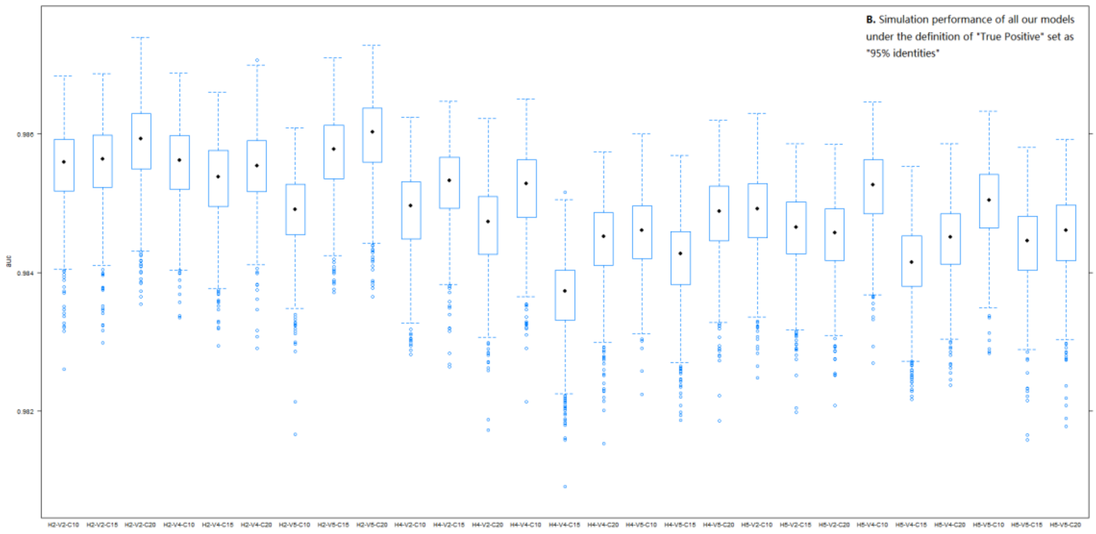
B. Simulation performance of all our models under the definition of "True Positive" set as "95% identities"
auc
H2-V2-C10 H2-V2-C15 H2-V2-C20 H2-V4-C10 H2-V4-C15 H2-V4-C20 H2-V5-C10 H2-V5-C15 H2-V5-C20 H4-V2-C10 H4-V2-C15 H4-V2-C20 H4-V4-C10 H4-V4-C15 H4-V4-C20 H4-V5-C10 H4-V5-C15 H4-V5-C20 H5-V2-C10 H5-V2-C15 H5-V2-C20 H5-V4-C10 H5-V4-C15 H5-V4-C20 H5-V5-C10 H5-V5-C15 H5-V5-C20

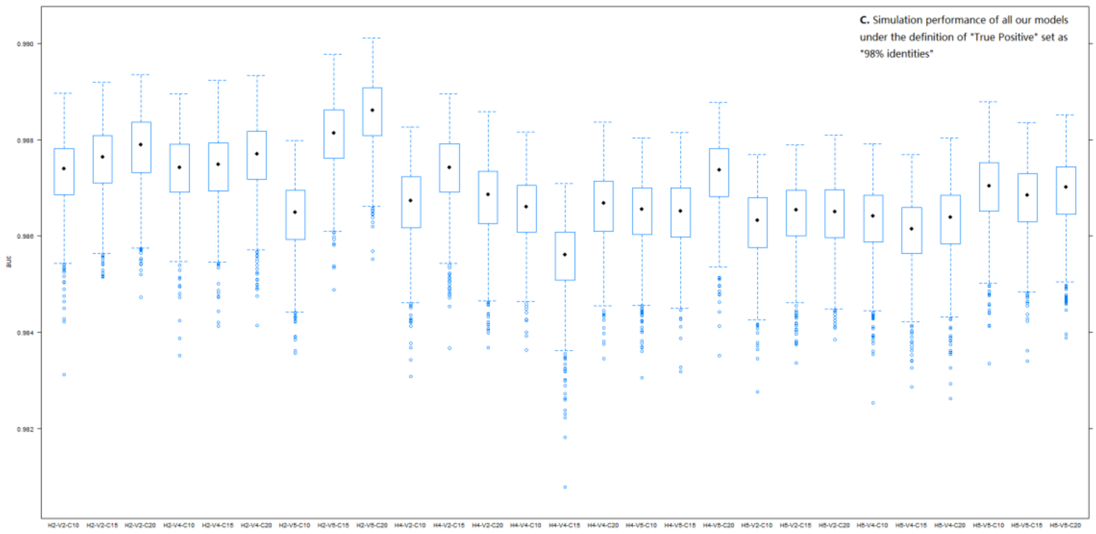
C. Simulation performance of all our models under the definition of "True Positive" set as "98% identities"
auc
H2-V2-C10 H2-V2-C15 H2-V2-C20 H2-V4-C10 H2-V4-C15 H2-V4-C20 H2-V5-C10 H2-V5-C15 H2-V5-C20 H4-V2-C10 H4-V2-C15 H4-V2-C20 H4-V4-C10 H4-V4-C15 H4-V4-C20 H4-V5-C10 H4-V5-C15 H4-V5-C20 H5-V2-C10 H5-V2-C15 H5-V2-C20 H5-V4-C10 H5-V4-C15 H5-V4-C20 H5-V5-C10 H5-V5-C15 H5-V5-C20

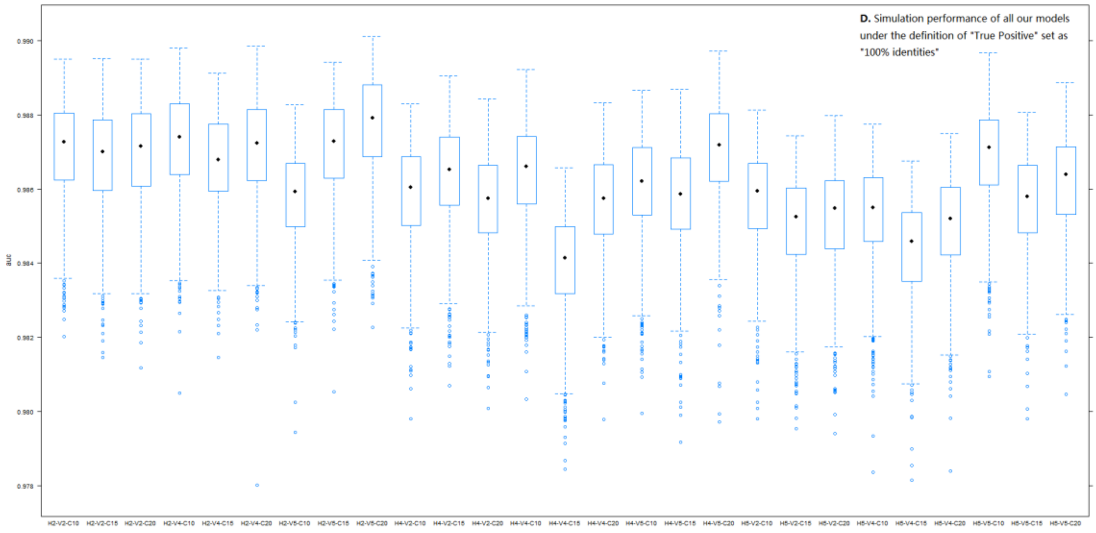
D. Simulation performance of all our models under the definition of "True Positive" set as "100% identities"

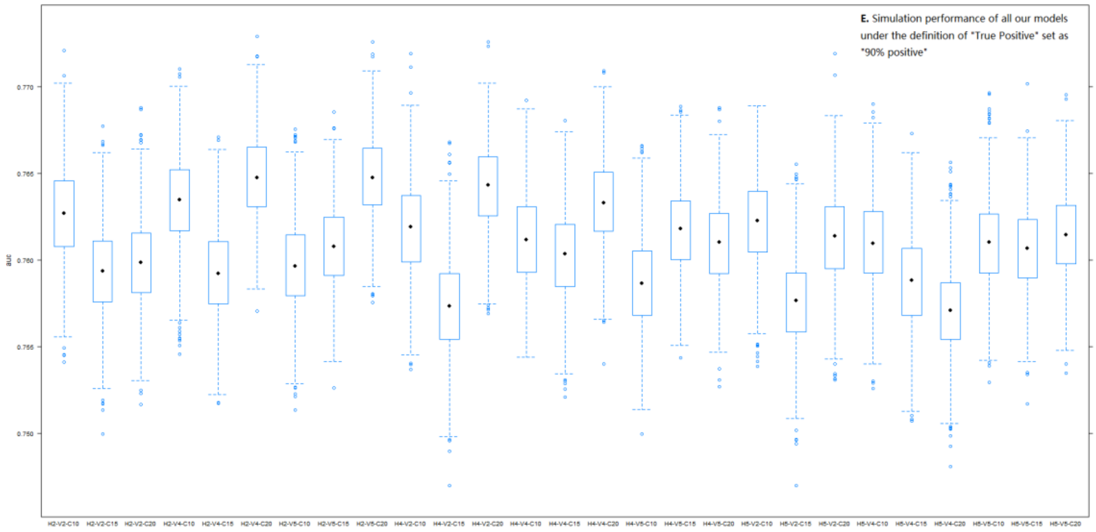
E. Simulation performance of all our models under the definition of "True Positive" set as "90% positive"

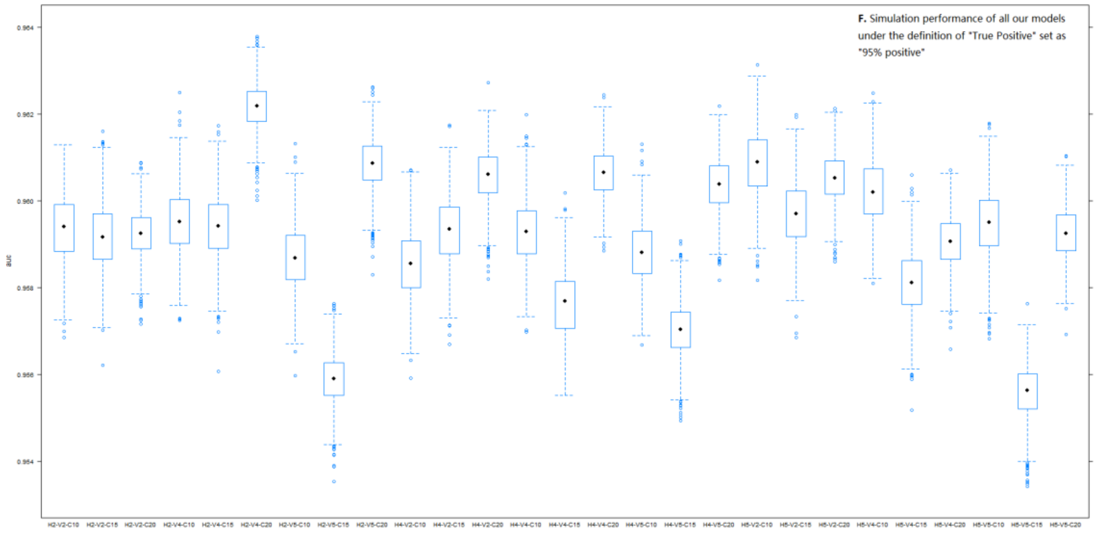
F. Simulation performance of all our models under the definition of "True Positive" set as "95% positive"

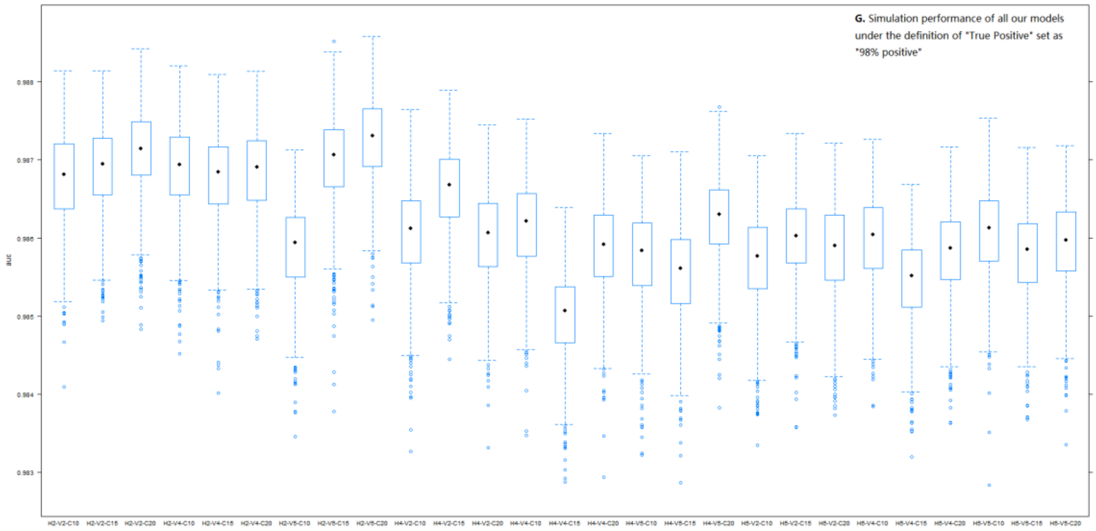

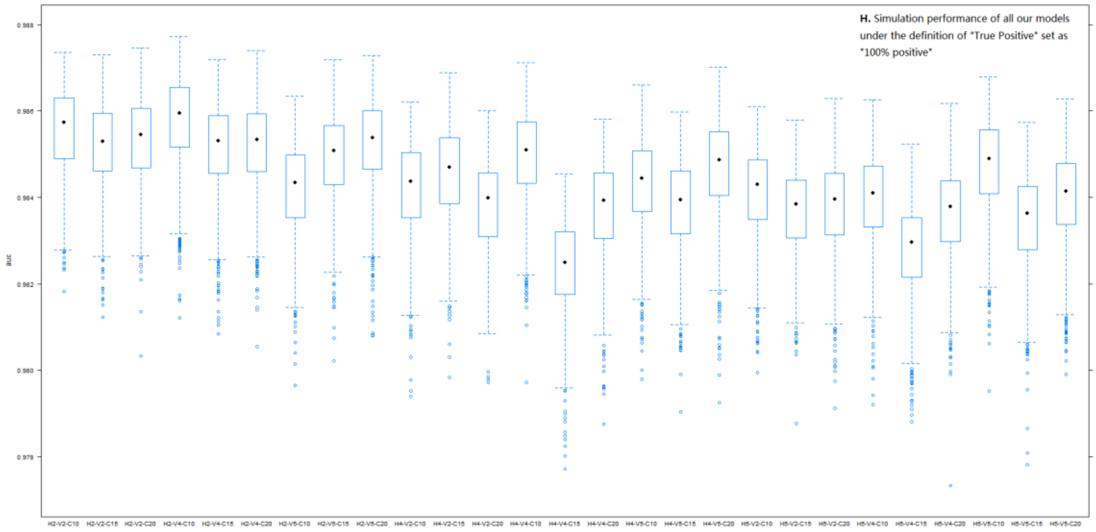

Supplementary Figure 11. Area Under Curve (AUC) value of 27 parameter combinations under eight different conditions generated by statistical simulation. Each time, positive samples were fixed and even numbers of negative samples were randomly selected to set up the simulation dataset. After 1000 times simulation for all parameter combinations and "True positive" conditions, AUC values and two-tail T-test were calculated between output scores of "Positive" samples and "Negative" samples. Eight Panels represent the simulation results of our methods and marked as Panel A~H respectively. Each Panel contains the results of 27 parameter combinations; one single boxplot contains 1000 points which generated by 1000 times of simulation by certain parameter combinations. Panels A~D presented results under the condition of "True positive" set as "90%, 95%, 98% and 100%" identities respectively; Panels E~H presented the results under the condition of "True positive" set as "90%, 95%, 98% and 100%" positive respectively ($P<2e^{-260}$). Positive samples contains 1.34% (90% identities), 1.05% (95% identities), 0.92% (98% identities), 0.7% (100% identities), 1.91% (90% positive), 1.21% (95% positive), 1.03% (98% positive) and 0.76% (100% positive) respectively.

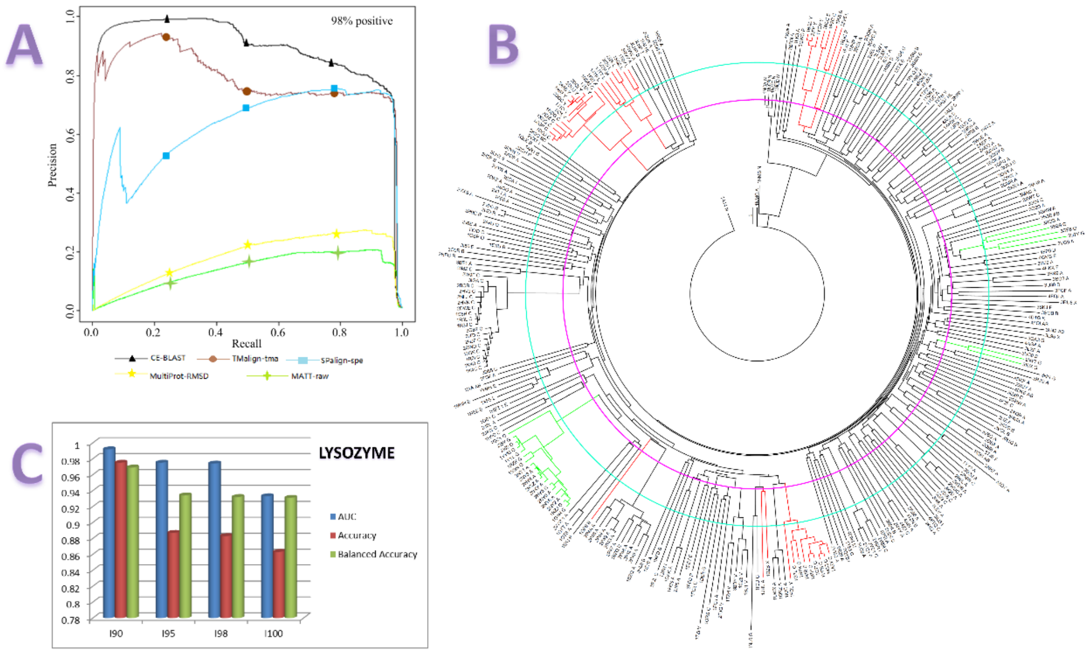

Supplementary Figure 12. Performance of CE-BLAST on 309 unique epitope structures derived from the PDB. (A) Performance comparison between CE-BLAST and other commonly used structural alignment tools on 95,172 pairs between 309 epitopes. The X-axis and Y-axis represent recall rate and precision, respectively, under a similarity cut-off of 98% positive in the V-region of the corresponding antibody. The result of CE-BLAST was obtained using the default parameter of H2-V2-C10. Then, the best results from peer tools were chosen. (B) Similarity clustering of 309 unique epitope structures by CE-BLAST. Red clusters represent the lysozyme epitope family, while green clusters represent that of gp120. The cyan circle indicates similarity score of 0.7, and the purple circle indicates that of 0.6. (C) Performance of CE-BLAST on 40 Lysozyme epitopes under different cut-offs, from 90% to 100% identities.

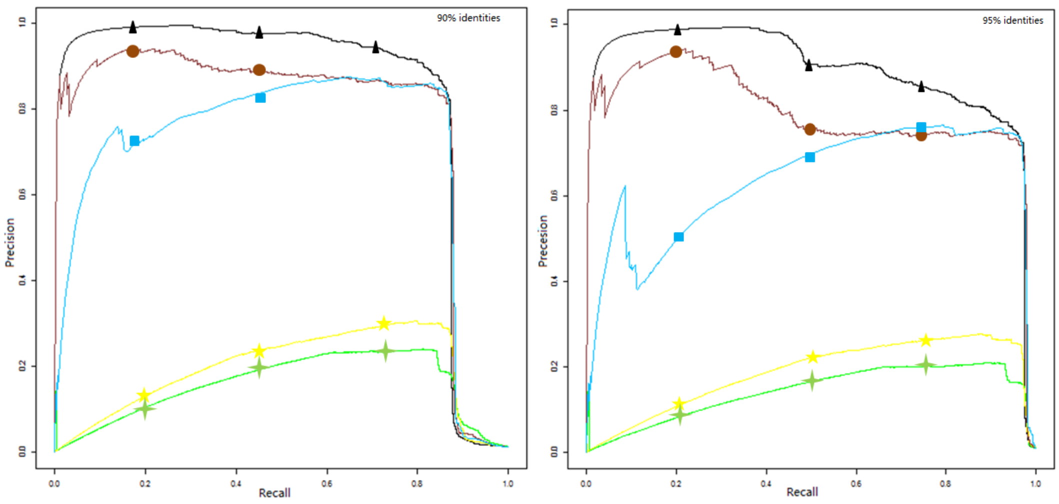

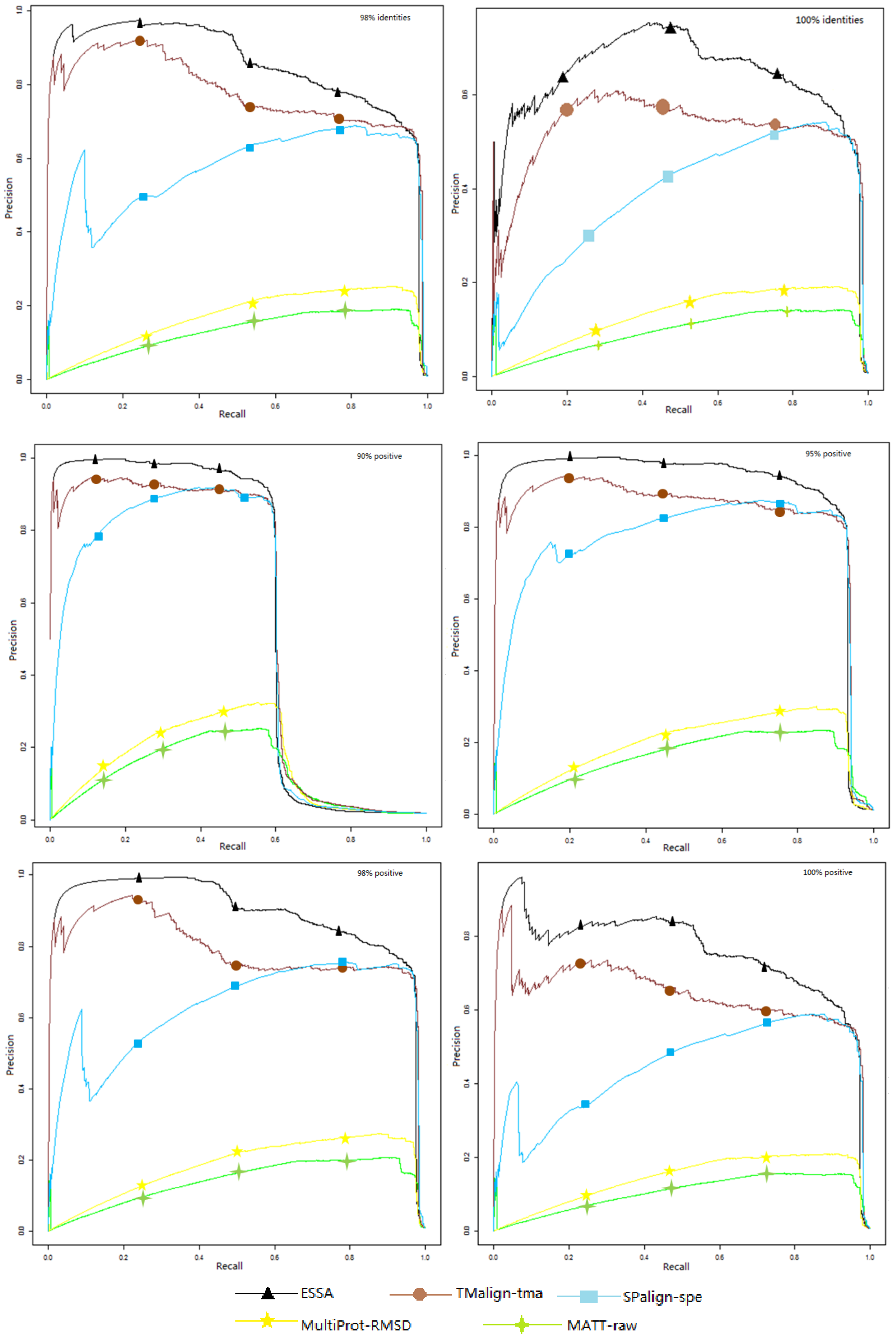

Supplementary Figure 13. Performance of CE-BLAST and compared peers in all eight conditions of "True positive". Eight Panels represent the results of our methods compared with peers on our entire dataset. Each Panel contains the best results of 5 algorithms under certain condition of "True positive". Top 4 panels presented results under the condition of "True positive" set as "90%, 95%, 98% and 100%" identities respectively; Last 4 panels presented the results under the condition of "True positive" set as "90%, 95%, 98% and 100%" positive respectively.

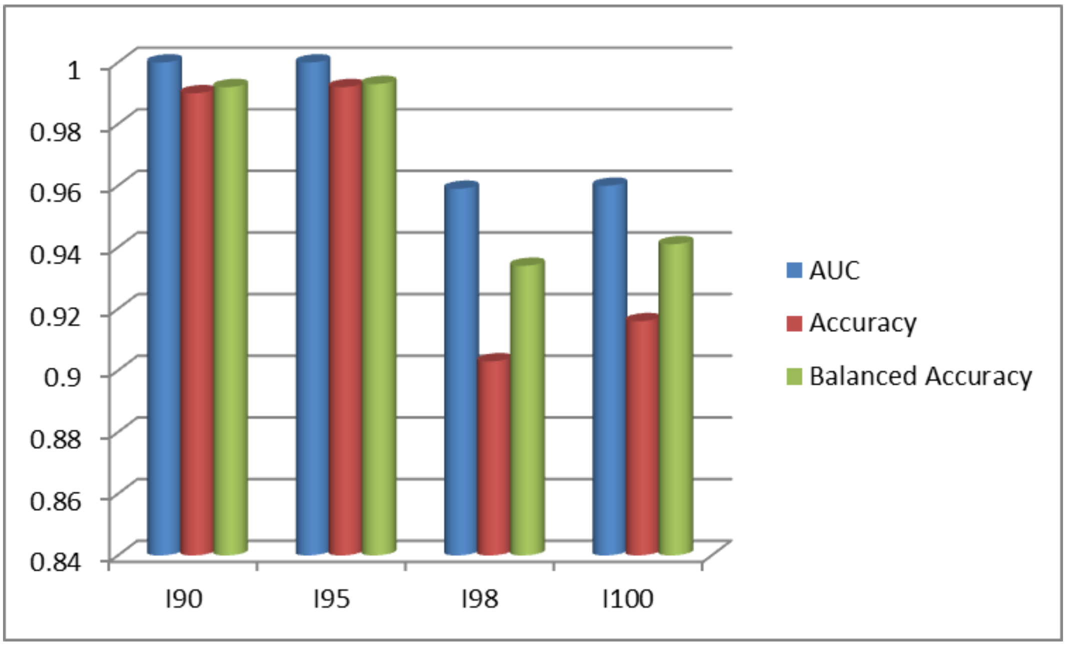

Supplementary Figure 14. Performance of CE-BLAST on 23 GP120 epitopes under different cutoffs from 90% to 100% identity.

Supplementary Tables

Supplementary Table 1. Official nomenclature ID and GenBank accession numbers of HA amino sequences of influenza viruses used in this study.

**Influenza viruses**	**HA Genbank ID**	**Abbreviation**
A/Moscow/10/1999(H3N2)	AAT08002	MO99 H3
A/Fujian/411/2002(H3N2)	ABB71825	FJ02 H3
A/Wisconsin/67/2005(H3N2)	ACF54576	WI05 H3
A/Brisbane/10/2007(H3N2)	ABW23353	BR07 H3
A/Hong Kong/1/1968(H3N2)	AFG71887	HK68 H3
A/Philippines/2/1982(H3N2))	ADJ41805	PH82 H3
A/Indiana/08/2011(H3N2))	AEO00689	IN11 H3

Supplementary Table 2. Neutralization titers of Con H3 immunized mice against different H3 viral strains.

		**Viral Strains**						
		WI05 H3	FJ02 H3	BR07 H3	MO99 H3	HK68 H3	PH82 H3	IN11 H3
**Con H3 immunized mice**	1	12150	800	3200	50	25	25	25
	2	36450	3200	6400	25	25	50	25
	3	4050	6400	800	400	25	25	25
	4	12150	800	3200	200	50	25	25
	5	12150	3200	3200	100	25	25	50
	GMT	12150	2111	2786	100	25	25	25
**Control group**	1	25	50	25	25	25	25	25
	2	25	50	25	25	25	25	50
	3	25	25	25	50	25	25	25
	GMT	25	37.5	25	35	25	25	35

Supplementary Table 3. Inhibition Concentration results of 7 tested strains to the monoclonal neutralization antibody of Con H3. IC 50 values were calculated by fitting.

**Tested strain**	**CE-BLAST***	**IC50**	**Antigenic similarity**
**A/Brisbane/10/2007**	2.46(+)	0.044 µ g/ml	Similar
**A/Wisconsin/67/2005**	2.5(+)	0.273 µ g/ml	Similar
**A/Fujian/411/2002**	3.99(+)	0.56 µ g/ml	Similar
**A/Moscow/10/1999**	17.25(+)	>100 µ g/ml	Escape

**A/Philippines/2/1982**	32.56(+)	>100 μ g/ml	Escape
**A/Hong Kong/1/1968**	182.7(+)	>100 μ g/ml	Escape
**A/Indiana/08/2011**	41.35(+)	>100 μ g/ml	Escape

*Predicted $log^{-1}D_{ab}$ (**Supplementary Note 2**) of our method compared with experimental values. $log^{-1}D_{ab} < 4$ indicate antigenic similar while $log^{-1}D_{ab} > 4$ indicate immune escape. (+) means matched prediction while (-) means wrong prediction.

Supplementary Table 4. Predicted results of 7 tested strains against Con H3 by Qiu's method [4].

Tested strain	1972	1977	1982	1987	1992	1997	2002	2007
**A/Brisbane/10/2007**	2.00(+)	17.96(-)	61.27(-)	5.64(-)	4.57(-)	4.17(-)	3.82(+)	1.83(+)
**A/Wisconsin/67/2005**	0.02(+)	11.50(-)	52.09(-)	38.11(-)	19.84(-)	8.49(-)	5.47(-)	1.68(+)
**A/Fujian/411/2002**	0.04(+)	19.07(-)	211.72(-)	75.06(-)	41.53(-)	18.23(-)	9.55(-)	3.09(+)
**A/Moscow/10/1999**	0.00(-)	1.89(-)	3.22(-)	186.63(+)	148.16(+)	43.93(+)	22.67(+)	21.14(+)
**A/Philippines/2/1982**	2.14(-)	7.40(+)	1628.13(+)	131.96(+)	99.39(+)	343.46(+)	654.84(+)	294.07(+)
**A/Hong Kong/1/1968**	2.60(-)	48.74(+)	0.85(-)	36.86(+)	9.58(+)	20.53(+)	47.97(+)	10.82(+)
**A/Indiana/08/2011**	17.17(+)	0.05(-)	3.36(-)	47.90(+)	705.74(+)	739.29(+)	1731.73(+)	653.48(+)
**wrong prediction**	3	5	6	3	3	3	2	0

(+) means matched prediction while (-) means wrong prediction.

Supplementary Table 5. Predicted results of 7 tested strains against Con H3 by Lees and Sheperd's method [5].

Tested strain	1972	1977	1982	1987	1992	1997	2002	2007
**A/Brisbane/10/2007**	6.22(-)	2.48(+)	2.44(+)	1.65(+)	2.09 (+)	2.16(+)	1.68(+)	1.16(+)
**A/Wisconsin/67/2005**	140.30(-)	9.97(-)	6.57(-)	6.57 (-)	6.72 (-)	7.82 (-)	7.35 (-)	3.91(+)
**A/Fujian/411/2002**	10.86(-)	6.92(-)	6.67(-)	6.16(-)	6.42 (-)	7.76 (-)	6.76 (-)	4.17 (-)
**A/Moscow/10/1999**	32.39(+)	79.65(+)	47.23(+)	39.44(+)	41.66(+)	43.10(+)	34.85(+)	39.06(+)
**A/Philippines/2/1982**	902.12(+)	551.84(+)	157.54(+)	105.83(+)	106.32(+)	82.57(+)	69.48(+)	120.08(+)
**A/Hong Kong/1/1968**	7403695.91(+)	6036.39(+)	297.41(+)	228.39(+)	207.53(+)	113.66(+)	118.71(+)	229.36 (+)
**A/Indiana/08/2011**	118391.98(+)	653.53(+)	55.07(+)	74.45(+)	79.42(+)	56.41(+)	39.98(+)	79.54(+)
**wrong prediction**	3	2	2	2	2	2	2	1

(+) means matched prediction while (-) means wrong prediction.

Supplementary Table 6. Predicted results of 7 tested strains against Con H3 by AntigenCO [6].

Tested strain	1972	1977	1982	1987	1992	1997	2002	2007
**A/Brisbane/10/2007**	0.7551 (+)	0.6371 (+)	1.2149 (+)	1.4111 (+)	1.1087 (+)	0.9861 (+)	1.2359 (+)	1.7515 (+)
**A/Wisconsin/67/2005**	0.9186 (+)	0.6673 (+)	1.7356 (+)	1.4111 (+)	1.1087 (+)	0.9861 (-)	1.2359 (+)	1.7515 (+)
**A/Fujian/411/2002**	0.9811 (+)	1.2327 (+)	2.3856 (-)	1.7507 (+)	1.8234 (+)	1.7057 (-)	2.0041 (-)	3.1901 (-)
**A/Moscow/10/1999**	0.7586 (-)	-0.0466 (-)	0.5221 (-)	1.3067 (-)	3.5968 (+)	4.3267 (+)	2.6150 (+)	2.8101 (+)
**A/Philippines/2/1982**	-1.4102 (-)	4.0246 (+)	4.2426 (+)	5.1267 (+)	6.5017 (+)	6.8682 (+)	6.4270 (+)	8.6698 (+)
**A/Hong Kong/1/1968**	-1.8001 (-)	0.9151 (-)	0.9418 (-)	6.3317 (+)	7.1024 (+)	8.3318 (+)	10.8465 (+)	12.6905 (+)
**A/Indiana/08/2011**	2.1159 (+)	2.0533 (+)	1.9634 (-)	2.8577 (+)	3.6610 (+)	4.1811 (+)	3.3795 (+)	4.0345 (+)
**wrong prediction**	3	2	4	1	0	0	1	1

(+) means matched prediction while (-) means wrong prediction.

Supplementary Table 7. Strain ID of DENV and ZIKV used to detect potential cross-reactive epitope

Strain Name	NCBI ID
DENV1/Cambodia/2003-BID-V1991	GQ868619
DENV1/Cambodia/2003-BID-V1995	FJ639680
DENV1/Malaysia/P72-1244-1/1972	AAN32776
DENV1/ NP_059433	NP_059433
DENV2/Cambodia/2007/BID-V4265	GU131927
DENV2/Malaysia/2008-DKD-811	FJ467493
DENV2/New Guinea/1944	AF038403
DENV2/Tonga/1974	AY744147
DENV3/Myanmar/2008	KT452792
DENV3/Philippines/H87/1956	P27915.1
DENV3/YP_001531168	YP_001531168.2
DENV3/YP_001621843	YP_001621843.1
DENV4/Brazil/2012/BR-12	KT452794
DENV4/Indonesia/1973/M30153/AC	KT452801
DENV4/Myanmar/2008/81087	KT452793
DENV4/Malaysia/1973/P73-1120	JF262780
ZIKV/China/2016	AMK79469
ZIKV/French Guiana/2016	AML81026
ZIKV/French Polynesia/2013	ANO46307
ZIKV/Francisco Morazán/2016	ARB07960

Supplementary Table 8. List of 309 epitope structures with PDB ids and chain names

PDB id	Chain	PDB id	Chain	PDB id	Chain	PDB id	Chain	PDB id	Chain
**1A14**	N	**1NDM**	C	**2BDN**	A	**2VH5**	R	**3LHP**	S
**1A2Y**	C	**1NFD**	B	**2BOB**	C	**2VOL**	B	**3LHP**	T
**1AHW**	C	**1NL0**	G	**2CMR**	A	**2VXQ**	A	**3LIZ**	A
**1AR1**	B	**1NMB**	N	**2DD8**	S	**2VXS**	A	**3LZF**	A
**1BJ1**	V	**1NMC**	A	**2DQC**	Y	**2VXT**	I	**3MA9**	A
**1BQL**	Y	**1NSN**	S	**2DQI**	Y	**2VYR**	A1	**3MAC**	A
**1BVK**	C	**1OAK**	A	**2DQJ**	Y	**2VYR**	A2	**3MJ9**	A
**1BZQ**	A	**1OAZ**	A	**2DWD**	C	**2W0F**	C	**3MXW**	A
**1BZQ**	D	**1OB1**	C	**2DWE**	C	**2W9E**	A	**3NFP**	I
**1CZ8**	V	**1OP9**	B	**2EIZ**	C	**2WUB**	A	**3NGB**	A
**1DEE**	G	**1ORS**	C	**2FD6**	U	**2WUC**	A	**3NH7**	A
**1DQJ**	C	**1OSP**	O	**2FJG**	V	**2XQB**	A	**3O0R**	B
**1DZB**	X	**1OTS**	A	**2GHW**	A	**2XQY**	A	**3O2D**	A
**1E6J**	P	**1OTT**	A	**2H9G**	R	**2XRA**	A	**3O8X**	A
**1EGJ**	A	**1P2C**	C	**2HFG**	R	**2XTJ**	A	**3O9W**	A
**1EO8**	A	**1PG7**	HL	**2HJF**	C	**2XWT**	C	**3PGF**	A
**1EXU**	B	**1PKQ**	E	**2HMI**	B	**2YBR**	C	**3PNW**	C
**1EZV**	E	**1QFU**	A	**2HVJ**	C	**2YBR**	I	**3Q1S**	I
**1FBI**	X	**1QKZ**	A	**2HVK**	C	**2YC1**	C	**3QA3**	G

**1FC2**	C	**1QLE**	B	**2I25**	L	**2YC1**	F	**3QWO**	P
**1FDL**	Y	**1R3I**	C	**2I26**	L	**2ZCH**	P	**3R1G**	B
**1FE8**	A	**1R3J**	C	**2I5Y**	G	**3A67**	Y	**3RJQ**	A
**1FJ1**	F	**1R3K**	C	**2I60**	G	**3A6B**	Y	**3RU8**	X
**1FNS**	A	**1R3L**	C	**2IFF**	Y	**3B2U**	A	**3RVV**	A
**1FSK**	A	**1RI8**	B	**2ITD**	C	**3B9K**	B	**3RVW**	A
**1G7I**	C	**1RJC**	B	**2IWG**	B	**3BDY**	V	**3SDY**	A
**1G7L**	C	**1RJL**	C	**2J4W**	D	**3BE1**	A	**3SDY**	B
**1G9M**	G	**1RZJ**	G	**2J5L**	A	**3BGF**	A	**3SE8**	G
**1G9N**	G	**1RZK**	G	**2J6E**	A	**3BN9**	A	**3SE9**	G
**1GC1**	G	**1S5H**	C	**2J88**	A	**3D85**	C	**3SKJ**	F
**1H0D**	C	**1SQ2**	L	**2JEL**	P	**3DVG**	X	**3SO3**	A
**1HEZ**	E1	**1SY6**	A	**2NLJ**	C	**3DVN**	V	**3SOB**	B
**1HEZ**	E2	**1T6V**	L	**2NR6**	A	**3EOA**	I	**3SQO**	A
**1I1A**	AB	**1T83**	C	**2NR6**	B	**3FFD**	P	**3T2N**	B
**1IAI**	HL	**1TPX**	A	**2NXY**	A	**3G04**	C	**3TJE**	F
**1IC5**	Y	**1TQB**	A	**2NXZ**	A	**3GB7**	C	**3U2S**	C
**1IGC**	A	**1TQC**	A	**2NY0**	A	**3GBM**	B	**3U30**	D
**1IQD**	C	**1TZH**	V	**2NY1**	A	**3GBN**	B	**3U7Y**	G
**1J1O**	Y	**1TZI**	V	**2NY2**	A	**3GI8**	C	**3UC0**	B
**1J1P**	Y	**1UJ3**	C	**2NY3**	A	**3GI9**	C	**3UX9**	C
**1JHL**	A	**1UWX**	A	**2NY4**	A	**3GRW**	A	**3V6O**	A
**1JPS**	T	**1V7M**	V	**2NY5**	G	**3H3B**	AB	**3VG9**	A
**1JRH**	I	**1VFB**	C	**2NY6**	A	**3H3P**	S	**4AEI**	A
**1JTP**	L	**1W72**	A	**2NY7**	G	**3H42**	AB	**4AEI**	C
**1JTP**	M	**1WEJ**	F	**2NYY**	A	**3HI1**	G	**4AG4**	A
**1JTT**	L	**1XF5**	L	**2P42**	A	**3HI6**	A	**4AL8**	C
**1K4C**	C	**1XGP**	C	**2P43**	A	**3HMX**	A	**4ALA**	C
**1K4D**	C	**1XGQ**	C	**2P44**	A	**3I50**	E	**4DGI**	A
**1KB5**	AB	**1XIW**	A	**2P45**	A	**3IDX**	G	**4DKE**	AB
**1KIP**	C	**1YJD**	C	**2P46**	A	**3IGA**	C	**4DKF**	A
**1KIQ**	C	**1YMH**	E	**2P47**	A	**3IU3**	I	**4DN4**	M
**1KIR**	C	**1YQV**	Y	**2P48**	A	**3K2U**	A	**4DTG**	K
**1LK3**	A	**1YY9**	A	**2P49**	A	**3K3Q**	BC	**4ETQ**	C
**1MHH**	E	**1YYL**	G	**2P4A**	A	**3KR3**	D	**4F2M**	E
**1MHP**	A	**1YYM**	G	**2Q8A**	A	**3KS0**	A	**4F2M**	F
**1MLC**	E	**1ZTX**	E	**2Q8B**	A	**3L5W**	I	**4F3F**	C
**1N8Z**	C	**1ZV5**	L	**2QQK**	A	**3L5X**	A	**4FP8**	D
**1NCA**	N	**2ADF**	A	**2QQN**	A	**3L5Y**	A	**4FQI**	AB
**1NCB**	N	**2AEP**	A	**2R0L**	A	**3L95**	X	**4FQJ**	A
**1NCC**	N	**2AEQ**	A	**2R29**	A	**3LD8**	A	**4GMS**	E
**1NCD**	N	**2ARJ**	Q	**2R56**	A	**3LEV**	A	**4HKX**	E
**1NDG**	C	**2B2X**	A	**2UZI**	R	**3LH2**	S		

Supplementary Table 9. Area Under Curve (AUC), True Positive Rate (TPR), Accuracy (ACC) and Balanced Accuracy (BA) of 27 parameter combinations under 8 different condition of "True positive".

**AUC**[a]	**I90***	**I95**	**I98**	**I100**	**P90**	**P95**	**P98**	**P100**
**h2.v2.c10**	0.892	0.958	0.96	0.951	0.761	0.932	0.962	0.952
**h2.v2.c15**	0.892	0.959	0.962	0.954	0.757	0.934	0.964	0.955
**h2.v2.c20**	0.896	0.954	0.957	0.949	0.77	0.932	0.959	0.95
**h2.v4.c10**	0.889	0.958	0.96	0.951	0.761	0.932	0.962	0.952
**h2.v4.c15**	0.89	0.956	0.96	0.95	0.756	0.932	0.962	0.951
**h2.v4.c20**	0.896	0.955	0.957	0.948	0.768	0.933	0.958	0.949
**h2.v5.c10**	0.889	0.958	0.96	0.951	0.759	0.932	0.962	0.952
**h2.v5.c15**	0.888	0.953	0.956	0.947	0.759	0.925	0.958	0.948
**h2.v5.c20**	0.893	0.953	0.956	0.947	0.766	0.93	0.958	0.948
**h4.v2.c10**	0.89	0.957	0.96	0.95	0.759	0.932	0.962	0.952
**h4.v2.c15**	0.892	0.959	0.963	0.954	0.757	0.935	0.965	0.955
**h4.v2.c20**	0.894	0.953	0.955	0.946	0.767	0.93	0.957	0.947
**h4.v4.c10**	0.891	0.958	0.96	0.951	0.759	0.934	0.963	0.953
**h4.v4.c15**	0.887	0.956	0.959	0.949	0.758	0.932	0.961	0.951
**h4.v4.c20**	0.895	0.954	0.956	0.948	0.767	0.931	0.958	0.949
**h4.v5.c10**	0.887	0.958	0.96	0.951	0.757	0.932	0.962	0.952
**h4.v5.c15**	0.891	0.953	0.957	0.949	0.769	0.927	0.959	0.95
**h4.v5.c20**	0.895	0.954	0.957	0.949	0.761	0.931	0.958	0.949
**h5.v2.c10**	0.892	0.958	0.96	0.951	0.76	0.935	0.963	0.953
**h5.v2.c15**	0.892	0.959	0.962	0.952	0.758	0.934	0.964	0.954
**h5.v2.c20**	0.893	0.953	0.955	0.946	0.766	0.931	0.957	0.947
**h5.v4.c10**	0.893	0.96	0.962	0.953	0.761	0.935	0.964	0.955
**h5.v4.c15**	0.889	0.957	0.961	0.951	0.757	0.932	0.963	0.952
**h5.v4.c20**	0.892	0.953	0.956	0.946	0.759	0.929	0.958	0.948
**h5.v5.c10**	0.89	0.958	0.961	0.952	0.76	0.933	0.963	0.953
**h5.v5.c15**	0.89	0.953	0.957	0.949	0.768	0.926	0.959	0.949
**h5.v5.c20**	0.893	0.954	0.957	0.949	0.763	0.93	0.959	0.949

**TPR**[b]	**I90**	**I95**	**I98**	**I100**	**P90**	**P95**	**P98**	**P100**
**h2.v2.c10**	0.811	0.915	0.92	0.901	0.594	0.878	0.924	0.903
**h2.v2.c15**	0.809	0.914	0.919	0.899	0.592	0.876	0.923	0.902
**h2.v2.c20**	0.809	0.912	0.915	0.895	0.606	0.876	0.92	0.897
**h2.v4.c10**	0.81	0.914	0.919	0.899	0.593	0.877	0.923	0.902
**h2.v4.c15**	0.808	0.914	0.919	0.899	0.592	0.876	0.923	0.902
**h2.v4.c20**	0.807	0.91	0.913	0.892	0.606	0.874	0.917	0.895
**h2.v5.c10**	0.808	0.913	0.918	0.901	0.591	0.876	0.922	0.903
**h2.v5.c15**	0.807	0.912	0.917	0.896	0.592	0.873	0.921	0.899
**h2.v5.c20**	0.807	0.91	0.913	0.892	0.606	0.873	0.918	0.895
**h4.v2.c10**	0.81	0.914	0.919	0.899	0.594	0.876	0.923	0.902
**h4.v2.c15**	0.809	0.913	0.918	0.898	0.591	0.876	0.922	0.9
**h4.v2.c20**	0.805	0.908	0.912	0.892	0.605	0.871	0.917	0.895

**h4.v4.c10**	0.807	0.913	0.918	0.898	0.591	0.875	0.922	0.9
**h4.v4.c15**	0.809	0.913	0.918	0.898	0.591	0.876	0.922	0.9
**h4.v4.c20**	0.805	0.909	0.913	0.892	0.604	0.873	0.918	0.895
**h4.v5.c10**	0.809	0.913	0.918	0.898	0.592	0.876	0.922	0.9
**h4.v5.c15**	0.805	0.907	0.907	0.887	0.606	0.867	0.916	0.89
**h4.v5.c20**	0.805	0.908	0.913	0.892	0.596	0.87	0.917	0.895
**h5.v2.c10**	0.809	0.913	0.918	0.898	0.592	0.875	0.922	0.9
**h5.v2.c15**	0.807	0.912	0.917	0.896	0.591	0.875	0.921	0.899
**h5.v2.c20**	0.807	0.91	0.913	0.893	0.603	0.873	0.918	0.896
**h5.v4.c10**	0.808	0.912	0.917	0.896	0.591	0.875	0.921	0.899
**h5.v4.c15**	0.808	0.912	0.917	0.896	0.591	0.874	0.921	0.899
**h5.v4.c20**	0.804	0.907	0.911	0.892	0.595	0.87	0.916	0.895
**h5.v5.c10**	0.81	0.914	0.919	0.899	0.592	0.877	0.923	0.902
**h5.v5.c15**	0.805	0.907	0.911	0.888	0.606	0.872	0.916	0.892
**h5.v5.c20**	0.803	0.908	0.912	0.892	0.601	0.869	0.917	0.892

**ACC**[c]	**I90**	**I95**	**I98**	**I100**	**P90**	**P95**	**P98**	**P100**
**h2.v2.c10**	0.994	0.994	0.993	0.991	0.989	0.995	0.994	0.992
**h2.v2.c15**	0.994	0.994	0.993	0.991	0.99	0.996	0.994	0.992
**h2.v2.c20**	0.981	0.982	0.981	0.978	0.977	0.982	0.982	0.979
**h2.v4.c10**	0.994	0.994	0.994	0.991	0.989	0.995	0.994	0.992
**h2.v4.c15**	0.994	0.993	0.993	0.99	0.988	0.995	0.993	0.991
**h2.v4.c20**	0.981	0.982	0.981	0.981	0.975	0.982	0.983	0.981
**h2.v5.c10**	0.994	0.994	0.993	0.988	0.99	0.995	0.994	0.989
**h2.v5.c15**	0.986	0.985	0.984	0.982	0.981	0.987	0.985	0.982
**h2.v5.c20**	0.982	0.983	0.983	0.981	0.975	0.984	0.984	0.982
**h4.v2.c10**	0.994	0.994	0.994	0.991	0.988	0.996	0.995	0.992
**h4.v2.c15**	0.993	0.994	0.993	0.991	0.989	0.995	0.994	0.992
**h4.v2.c20**	0.983	0.983	0.982	0.981	0.976	0.984	0.983	0.981
**h4.v4.c10**	0.995	0.994	0.993	0.991	0.99	0.995	0.994	0.991
**h4.v4.c15**	0.993	0.994	0.993	0.991	0.989	0.995	0.994	0.992
**h4.v4.c20**	0.983	0.981	0.98	0.981	0.976	0.982	0.981	0.981
**h4.v5.c10**	0.994	0.995	0.994	0.992	0.989	0.995	0.995	0.992
**h4.v5.c15**	0.981	0.981	0.984	0.982	0.976	0.985	0.981	0.982
**h4.v5.c20**	0.985	0.985	0.982	0.982	0.979	0.985	0.985	0.982
**h5.v2.c10**	0.994	0.995	0.994	0.992	0.989	0.995	0.995	0.992
**h5.v2.c15**	0.994	0.995	0.994	0.992	0.989	0.995	0.995	0.992
**h5.v2.c20**	0.98	0.981	0.981	0.979	0.977	0.982	0.982	0.98
**h5.v4.c10**	0.994	0.995	0.994	0.992	0.99	0.995	0.995	0.992
**h5.v4.c15**	0.994	0.995	0.994	0.992	0.99	0.996	0.995	0.992
**h5.v4.c20**	0.985	0.985	0.984	0.983	0.979	0.985	0.985	0.983
**h5.v5.c10**	0.993	0.994	0.993	0.991	0.989	0.995	0.994	0.991
**h5.v5.c15**	0.981	0.981	0.98	0.98	0.977	0.979	0.981	0.98
**h5.v5.c20**	0.983	0.981	0.98	0.978	0.977	0.984	0.981	0.981

BA[d]	I90	I95	I98	I100	P90	P95	P98	P100
**h2.v2.c10**	0.904	0.955	0.957	0.946	0.795	0.937	0.959	0.948
**h2.v2.c15**	0.903	0.955	0.956	0.946	0.795	0.936	0.959	0.947
**h2.v2.c20**	0.896	0.947	0.948	0.937	0.795	0.929	0.951	0.938
**h2.v4.c10**	0.903	0.955	0.957	0.946	0.795	0.936	0.959	0.947
**h2.v4.c15**	0.902	0.954	0.956	0.945	0.794	0.936	0.959	0.947
**h2.v4.c20**	0.895	0.946	0.947	0.936	0.794	0.929	0.95	0.938
**h2.v5.c10**	0.902	0.954	0.956	0.945	0.794	0.936	0.958	0.946
**h2.v5.c15**	0.898	0.949	0.95	0.939	0.791	0.931	0.953	0.941
**h2.v5.c20**	0.896	0.947	0.948	0.937	0.794	0.929	0.951	0.938
**h4.v2.c10**	0.903	0.955	0.957	0.946	0.795	0.936	0.959	0.947
**h4.v2.c15**	0.902	0.954	0.956	0.945	0.794	0.936	0.958	0.946
**h4.v2.c20**	0.895	0.946	0.947	0.936	0.794	0.928	0.95	0.938
**h4.v4.c10**	0.902	0.954	0.956	0.945	0.794	0.936	0.958	0.946
**h4.v4.c15**	0.902	0.954	0.956	0.945	0.794	0.936	0.958	0.946
**h4.v4.c20**	0.895	0.945	0.947	0.936	0.793	0.928	0.95	0.938
**h4.v5.c10**	0.903	0.954	0.956	0.945	0.794	0.936	0.959	0.947
**h4.v5.c15**	0.894	0.944	0.946	0.935	0.795	0.926	0.948	0.937
**h4.v5.c20**	0.896	0.947	0.948	0.937	0.791	0.929	0.951	0.939
**h5.v2.c10**	0.903	0.954	0.956	0.945	0.794	0.936	0.959	0.947
**h5.v2.c15**	0.902	0.954	0.956	0.944	0.794	0.935	0.958	0.946
**h5.v2.c20**	0.895	0.946	0.947	0.937	0.794	0.928	0.95	0.938
**h5.v4.c10**	0.902	0.954	0.956	0.944	0.794	0.936	0.958	0.946
**h5.v4.c15**	0.902	0.954	0.956	0.944	0.794	0.936	0.958	0.946
**h5.v4.c20**	0.895	0.946	0.947	0.937	0.791	0.928	0.95	0.939
**h5.v5.c10**	0.903	0.954	0.956	0.945	0.794	0.936	0.959	0.947
**h5.v5.c15**	0.894	0.944	0.946	0.934	0.795	0.926	0.949	0.936
**h5.v5.c20**	0.894	0.945	0.946	0.935	0.793	0.927	0.949	0.937

*"I" means sequence identities of v-region and "P" means sequence positives of v-region in BLAST. For example, "I90" means under the condition of "True positive" defined as sequence similarity over 90% for identities in v-region. The threshold of ROC was set as the point most close to (0, 1)

[a] AUC value means the area under the receiver operating characteristic curve, range from 0 to 1.

[b] TPR is defined as (True positive)/(True positive + False negative).

[c] ACC is defined as (True positive + True negative)/(Positive + Negative).

[d] BA is defined as (0.5 * True positive)/(True positive + False negative) + (0.5 *True negative)/(True negative + False positive)

Supplementary Table 10. Performance for different parameter combinations.*

AUC	I90	I95	I98	I100	P90	P95	P98	P100
**c10**	0.890333	0.958111	0.960333	0.951222	0.759667	0.933	0.962556	0.952667
**c15**	0.890111	0.956111	0.959667	0.950556	0.759889	0.930778	0.961667	0.951667
**c20**	0.894111	0.953667	0.956222	0.947556	0.765222	0.930778	0.958	0.948444
**AUC**	**I90**	**I95**	**I98**	**I100**	**P90**	**P95**	**P98**	**P100**
**h2.v2**	0.893333	0.957	0.959667	0.951333	0.762667	0.932667	0.961667	0.952333
**h2.v4**	0.891667	0.956333	0.959	0.949667	0.761667	0.932333	0.960667	0.950667

**h2.v5**	0.89	0.954667	0.957333	0.948333	0.761333	0.929	0.959333	0.949333
**h4.v2**	0.925333	0.970333	0.972333	0.965333	0.835333	0.954	0.974	0.966333
**h4.v4**	0.891	0.956	0.958333	0.949333	0.761333	0.932333	0.960667	0.951
**h4.v5**	0.891	0.955	0.958	0.949667	0.762333	0.93	0.959667	0.950333
**h5.v2**	0.892333	0.956667	0.959	0.949667	0.761333	0.933333	0.961333	0.951333
**h5.v4**	0.891333	0.956667	0.959667	0.95	0.759	0.932	0.961667	0.951667
**h5.v5**	0.891	0.955	0.958333	0.95	0.763667	0.929667	0.960333	0.950333

*"c10~c20" means the average AUC of those parameter combinations selected cutoff of searching granularity as 10Å, 15Å and 20Å (Compared with the cutoff of 20Å, the cutoff of 15Å requires about 3.8 times the time while 10Å requires about 7.1 times respectively). "h2.v2~h5.v5" means the average AUC of those models selected traverse all pixel combinations.

Supplementary Table 11. The top CE-BLAST searching results for a query of lysozyme epitope (PDB ID 1J1O_Y). The epitope difference illustrates the compositional difference in comparison to the query epitope. The V-similarity represents the sequence similarity of the corresponding antibody in immune complexes in the form of positive score / identity score.

Rank	Epitope ID in PDB	Epitope difference	V-similarity (positive/identity)	Similarity score of CE-BLAST
**No.1**	1J1O_Y	None	100%/100%	1.0000
**No.2**	2DQJ_Y	None	100%/99.5%	0.9908
**No.3**	3A67_Y	None	100%/99%	0.9892
**No.4**	2DQC_Y	None	100%/99%	0.9891
**No.5**	1J1P_Y	None	100%/99%	0.9870
**No.6**	2DQI_Y	None	99.5%/99.5%	0.9775
**No.7**	3A6B_Y	None	100%/99%	0.9755
**No.8**	1IC5_Y	None	99%/99%	0.9744
**No.9**	1NDM_C	1del	97%/94%	0.8832
**No.10**	1DQJ_C	1del	97%/93%	0.8781
**No.11**	1XGQ_C	1del	96%/92%	0.8735
**No.12**	1NDG_C	2del	97%/92%	0.8633
**No.13**	1XGP_C	2del	96%/92%	0.8105
**No.14**	2EIZ_C	2del	90%/77%	0.7849
**No.15**	1FBI_X	84% overlapping	69%/50%	0.6949
**No. 16**	1DZB_X	55% overlapping	61%/47%	0.5308

Supplementary Table 12. Top 15 hit epitope of query 1J1O_Y with residual composition and corresponding antibody similarity information. Epitope structures are ranked as descending sort according to our scores (H2-V2-C10), antibody similarity represents the similarity level of the corresponding antibodies' V-region.

**Ranking**	**Epitope file**	**epitope composition**
No.1	1J1O_Y.epi	13-22：LYS-ARG-HIS-GLY-LEU-ASP-ASN-TYR-ARG-GLY 62-63：TRP-TRP 72-77：SER-ARG-ASN-LEU-CYS-ASN 89：THR 92-93：VAL-ASN<br>96-104：LYS-LYS-ILE-VAL-SER-ASP-GLY-ASN-GLY 107：ALA
No.2	2DQJ_Y.epi	13-22：LYS-ARG-HIS-GLY-LEU-ASP-ASN-TYR-ARG-GLY 62-63：TRP-TRP<br>72-77：SER-ARG-ASN-LEU-CYS-ASN 89：THR 92-93：VAL-ASN<br>96-104：LYS-LYS-ILE-VAL-SER-ASP-GLY-ASN-GLY 107：ALA

**No.**3	3A67_Y.epi	13-22：LYS-ARG-HIS-GLY-LEU-ASP-ASN-TYR-ARG-GLY 62-63：TRP-TRP<br>72-77：SER-ARG-ASN-LEU-CYS-ASN 89：THR 92-93：VAL-ASN<br>96-104：LYS-LYS-ILE-VAL-SER-ASP-GLY-ASN-GLY 107：ALA
No.4	2DQC_Y.epi	13-22：LYS-ARG-HIS-GLY-LEU-ASP-ASN-TYR-ARG-GLY 62-63：TRP-TRP<br>72-77：SER-ARG-ASN-LEU-CYS-ASN 89：THR 92-93：VAL-ASN<br>96-104：LYS-LYS-ILE-VAL-SER-ASP-GLY-ASN-GLY 107：ALA
No.5	1J1P_Y.epi	13-22：LYS-ARG-HIS-GLY-LEU-ASP-ASN-TYR-ARG-GLY 62-63：TRP-TRP<br>72-77：SER-ARG-ASN-LEU-CYS-ASN 89：THR 92-93：VAL-ASN<br>96-104：LYS-LYS-ILE-VAL-SER-ASP-GLY-ASN-GLY 107：ALA
No.6	2DQI_Y.epi	13-22：LYS-ARG-HIS-GLY-LEU-ASP-ASN-TYR-ARG-GLY 62-63：TRP-TRP<br>72-77：SER-ARG-ASN-LEU-CYS-ASN 89：THR 92-93：VAL-ASN<br>96-104：LYS-LYS-ILE-VAL-SER-ASP-GLY-ASN-GLY 107： ALA
No.7	3A6B_Y.epi	13-22：LYS-ARG-HIS-GLY-LEU-ASP-ASN-TYR-ARG-GLY 62-63：TRP-TRP<br>72-77：SER-ARG-ASN-LEU-CYS-ASN 89：THR 92-93：VAL-ASN<br>96-104：LYS-LYS-ILE-VAL-SER-ASP-GLY-ASN-GLY 107：ALA
No.8	1IC5_Y.epi	13-22：LYS-ARG-HIS-GLY-LEU-ASP-ASN-TYR-ARG-GLY 62-63：TRP-TRP<br>72-77：SER-ARG-ASN-LEU-CYS-ASN 89：THR 92-93：VAL-ASN<br>96-104：LYS-LYS-ILE-VAL-SER-ASP-GLY-ASN-GLY 107：ALA
No.9	1NDM_C.epi	13-22：LYS-ARG-HIS-GLY-LEU-ASP-ASN-TYR-ARG-GLY 62-63：TRP-TRP<br>73-77：ARG-ASN-LEU-CYS-ASN 89：THR 92-93：VAL-ASN<br>96-104：LYS-LYS-ILE-VAL-SER-ASP-GLY-ASN-GLY 107：ALA
No.10	1DQJ_C.epi	13-22：LYS-ARG-HIS-GLY-LEU-ASP-ASN-TYR-ARG-GLY 62-63：TRP-TRP<br>72-75：SER-ARG-ASN-LEU 77：ASN 89：THR 92-93：VAL-ASN<br>96-104：LYS-LYS-ILE-VAL-SER-ASP-GLY-ASN-GLY 107：ALA
No.11	1XGQ_C.epi	13-22：LYS-ARG-HIS-GLY-LEU-ASP-ASN-TYR-ARG-GLY 62-63：TRP-TRP<br>72-75：SER-ARG-ASN-LEU 77：ASN 89：THR 92-93：VAL-ASN<br>96-104：LYS-LYS-ILE-VAL-SER-ASP-GLY-ASN-GLY 107：ALA
No.12	1NDG_C.epi	13-22：LYS-ARG-HIS-GLY-LEU-ASP-ASN-TYR-ARG-GLY 62-63：TRP-TRP<br>72-77：SER-ARG-ASN-LEU-CYS-ASN 89：THR 92-93：VAL-ASN<br>96-104：LYS-LYS-ILE-VAL-SER-ASP-GLY-ASN
No.13	1XGP_C.epi	13-22：LYS-ARG-HIS-GLY-LEU-ASP-ASN-TYR-ARG-GLY 62-63：TRP-TRP<br>73-75：ARG-ASN-LEU 77：ASN 89：THR<br>92-93：VAL-ASN 96-104：LYS-LYS-ILE-VAL-SER-ASP-GLY-ASN-GLY
No.14	2EIZ_C.epi	13-22：LYS-ARG-HIS-GLY-LEU-ASP-ASN-TYR-ARG-<br>GLY 62-63：TRP-TRP 72-75：SER-ARG-ASN-LEU 77：ASN 89：THR<br>92-93：VAL-ASN 96-104：LYS-LYS-ILE-VAL-SER-ASP-GLY-ASN-GLY
No.15	1FBI_X.epi	14-17：ARG-HIS-GLY-LEU 19-22：ASN-TYR-ARG-GLY 62-63：TRP-TRP 71-78：<br>GLY-SER-ARG-ASN-LEU-CYS-ASN-ILE 89-90：THR-ALA 92-94：ALA-ASN-CYS<br>100-102：SER-ASP-GLY

Supplementary Table 13. Top ranking list of similar epitopes for a GP120 epitopes (PDB: 2NXZ_A as query) from CE-BLAST.

Rank	PDB ID	Epitope difference[a]	V-similarity P/I[b]	CE-BLAST
**No.1**	2NXZ_A	None	100%/100%	1

**No.2**	2NY4_A	None	100%/100%	0.9901
**No.3**	2NY3_A	None	100%/100%	0.9844
**No.4**	2NY1_A	None	100%/100%	0.9766
**No.5**	2NXY_A	None	100%/100%	0.9743
**No.6**	2NY2_A	1add	100%/100%	0.9736
**No.7**	2NY0_A	1mutation	100%/100%	0.9704
**No.8**	2NY5_G	1mutation	100%/100%	0.9649
**No.9**	2NY6_A	1add	100%/100%	0.954
**No.10**	1RZJ_G	1add	100%/100%	0.9303
**No.11**	1G9M_G	1add	99%/97.5%	0.927
**No.12**	1G9N_G	1add	99%/97.5%	0.9078
**No.13**	2I60_G	1add	100%/100%	0.8987
**No.14**	1RZK_G	2add	100%/100%	0.8961
**No.15**	1YYL_G	1add	100%/100%	0.8531
**No.16**	2I5Y_G	1add	100%/100%	0.8119
**No.17**	1GC1_G	1add/1del	99.5%/97.5%	0.7827
**Under threshold**				
**No.18**	2NY7_G	10% overlap	73.5%/61.5%	0.387
**No.19**	3IDX_G	15% overlap	76.5%/60.5%	0.3579

[a]Epitope differences illustrated the compositional difference to the query epitope. [b]V-similarity represents the antigenicity variation in terms of V-region sequence similarity of the corresponding antibody in immune-complexes.

Supplementary Notes

Supplementary Note 1. High performance on known conformational epitopes

CE-BLAST was first validated on 309 non-redundant known conformational epitopes from 298 antigen-antibody complexes in the PDB database. The PDB ID and corresponding chain information can be found in **Supplementary Table 8**. The preferable standard of antigenicity similarity would be derived from a series of antibody or antiserum cross-reactivity data such as HI assays for influenza virus. Due to the data scarcity of the cross-reactivity between antigens in immune complexes, we used the sequence similarity of the variable (V) regions of the corresponding antibodies as a measurement of antigenicity similarity between epitopes. To check its qualification as a quantifier of antigenicity similarity, the sequence similarity of the (V) regions of antibodies were examined in correlation with the variance of epitope compositions. Lysozyme was chosen as an example under test since it is the largest protein family in our dataset.

There are total 40 lysozyme-antibody complexes in PDB. The size of lysozyme epitopes is on average of 26±4. The Pearson correlation coefficient between epitope compositional difference and V-region similarity of corresponding antibodies can reach 0.7346 (identities). From the literature recording, antibodies are derived from different immune host such as mice (28 complexes), camelus, gallus and human. Considering the V-sequence bias of different immune host, we take the 28 complexes from mice for further examine. The correlation between epitope compositional difference and the V-region sequence similarity was increased to 0.8725 (antibody positive similarity) and 0.8666 (antibody identity similarity), as being shown in **Supplementary Fig. 10**. The high correlation above suggests the feasibility of using V-region sequence similarity of antibodies as a measurement of antigenicity similarity. However, we also noted that the same conformational epitope of lysozyme may induce quite different V-region antibodies from different immune host. For instance, the sequence identify score of V-region is only 75% between human antibody (2EIZ) and mice antibody (1XGP) to the same lysozyme epitope. This seems to indicate that it is more meaningful to adopt V-region sequence similarity of antibodies as a measurement of antigenicity similarity in the case of same immune host.

In that case, values of 100%, 98%, 95% and 90% of V-region identity/similarity were each tested as functional similarity cut-offs for the corresponding epitopes. A pair of epitopes is considered a true positive for similar antigenicity if the identity/similarity score of their

corresponding V-regions is above the cut-off, and vice versa. Then, the AUC (Area Under the Curve) value of the ROC (Receiver Operating Characteristic) curve, true positive rate, accuracy, and balanced accuracy were calculated (**Supplementary Table 9**). Different resolutions of the spin-image and the searching granularity of neighbouring residues were also scanned to identify an optimized parameter combination to balance the computational time and the accuracy (**Supplementary Table 10**). A horizontal pixel (H) value of 2Å, a vertical pixel (V) value of 2Å, and searching granularity of 10Å (H2-V2-C10) were set as default in our program, with 230 digits of fingerprint for each residue. When setting the true positive cut-off as 100% V-region identity, CE-BLAST can give an overall AUC value above 0.95; when using 98% V-region identity as the cut-off, CE-BLAST gives an AUC value of 0.96, indicating the outstanding ability of CE-BLAST to compare epitope similarities. Considering the potential bias caused by the unbalanced dataset, random sampling was performed under different parameter combinations with the same number of positive and negative samples. CE-BLAST achieved an AUC value of over 0.978 under all cut-offs of 98% positive/identities or 100% positive/identities (**Supplementary Fig. 11**), suggesting that the unbalanced dataset is not likely to influence the performance of CE-BLAST.

As the first tailor-made program to align structural epitopes, the performance of CE-BLAST was compared with that of commonly used structural alignment tools: MultiProt[7], MATT[8], TMalign[9] and SPalign [10]. As most of these peer programs provide several scores under different parameters, the best result from each program on the total of 95,172 epitope pairs was plotted in **Supplementary Fig. 12A** with precision and recall rate. Further detailed results for the different cut-offs can be found in **Supplementary Fig. 13**. According to the evaluation criteria, a good model would be able to maintain a consistently high precision as the recall rate increases[11]. As shown, CE-BLAST consistently maintains the highest precision level among all peer programs under different recall rates.

Because of the inherent sensitivity of the conformational epitopes, we tested CE-BLAST on mutant epitopes of protein antigens. All 309 unique epitopes were clustered according to the CE-BLAST similarity score as shown in **Supplementary Fig. 12B**. Further analyses revealed that lysozyme epitopes are the most abundant, with 40 structures, and that HIV gp120 epitopes are the second most abundant, with 23 structures. Interestingly, several sub-clusters were identified for both the lysozyme epitopes and the gp120 epitopes in **Supplementary Fig. 12B**, indicating

mutated, overlapping or unique epitopes. **Supplementary Fig. 12C** illustrates the performance of CE-BLAST on the lysozyme family, with an AUC value above 0.92, an accuracy value above 0.85, and a balanced accuracy value above 0.92 under different V-region similarity cut-off values. The results for gp120 can be found in **Supplementary Fig. 14**. According to background data from the PDB, a similarity score over 0.7 is empirically derived to select highly similar epitopes, a score below 0.6 indicates different epitopes, and a score in between these two values often indicates similar or partially overlapping epitopes.

We show more detailed results for lysozyme as an example. Using the lysozyme epitope 1J1O_Y (PDB id: 1J1O; Chain Y) as a random query, the similar epitopes are ranked according to similarity score in **Supplementary Table 11**, together with their sequence similarity for the V-region of the corresponding antibodies. The top 8 epitope structures are actually the same epitope, followed by 6 highly similar epitopes with one or two residue deletions, then by 2 partially overlapping epitopes (**Supplementary Table 12**). Similar results were observed for the gp120 epitopes (**Supplementary Table 13**). As displayed, the CE-BLAST score agrees well with the sequence difference of the V region in the corresponding antibodies. The above results suggest the outstanding performance of CE-BLAST both in a large testing dataset across antigen class and in a specific class of antigen mutants. All of the 559 epitope structures derived from immune complexes in the PDB have been combined into a built-in epitope database for the user to search against in CE-BLAST.

Supplementary Note 2. Data Collection

2.1 HA1 sequence collection

(1) NCBI Influenza Virus Database (http://www.ncbi.nlm.nih.gov/genomes/FLU/Database/)

(2) Global Initiative on Sharing All Influenza Data (http://platform.gisaid.org/)

(3) Squires, R.B., et al. (2012) Influenza research database: an integrated bioinformatics resource for influenza research and surveillance, Influenza and other respiratory viruses, 6, 404-416.

(4) Simon, C., et al. (2015) FluKB: A Knowledge-Based System for Influenza Vaccine Target Discovery and Analysis of the Immunological Properties of Influenza Viruses, Journal of immunology research, 2015, 380975.

(5) reports from National Institute for Medical Research (NIMR) (http://www.nimr.mrc.ac.uk/)

2.2 HI assay data collection

(1) Organization: U.S. Food and Drug Administration.

Access:

http://www.fda.gov/AdvisoryCommittees/CommitteesMeetingMaterials/BloodVaccinesandOt

herBiologics/VaccinesandRelatedBiologicalProductsAdvisoryCommittee/default.htm.

Detail:

Information for the Vaccines and Related Biological Products Advisory Committee. February 22, 2010

Information for the Vaccines and Related Biological Products Advisory Committee. (Seasonal Influenza Vaccines).February 25, 2011

Information for the Vaccines and Related Biological Products Advisory Committee. (Seasonal Influenza and Zoonotic Influenza). February 28, 2012

Information for the Vaccines and Related Biological Products Advisory Committee. (Information Regarding Seasonal Influenza Viruses). February 27, 2013

(2) Organization: World Health Organization.

Access: http://www.who.int/wer/en/

Detail:

Weekly Epidemiological Record 1969 No.1 ~ No.51-52
Weekly Epidemiological Record 1970 No.1-2 ~ No.52
Weekly Epidemiological Record 1971 No.1-2 ~ No.52-53
Weekly Epidemiological Record 1972 No.1 ~ No.51-52
Weekly Epidemiological Record 1973 No.1 ~ No.52
Weekly Epidemiological Record 1974 No.1 ~ No.51-52
Weekly Epidemiological Record 1975 No.1-2 ~ No.52
Weekly Epidemiological Record 1976 No.1-2 ~ No.52
Weekly Epidemiological Record 1977 No.1 ~ No.52
Weekly Epidemiological Record 1978 No.1 ~ No.51-52
Weekly Epidemiological Record 1979 No.1 ~ No.51-52
Weekly Epidemiological Record 1980 No.1 ~ No.52
Weekly Epidemiological Record 1981 No.1 ~ No.51-52
Weekly Epidemiological Record 1982 No.1 ~ No.51-52
Weekly Epidemiological Record 1983 No.1 ~ No.51-52
Weekly Epidemiological Record 1984 No.1 ~ No.51-52
Weekly Epidemiological Record 1985 No.1 ~ No.51-52
Weekly Epidemiological Record 1986 No.1 ~ No.51-52
Weekly Epidemiological Record 1987 No.1-2 ~ No.51-52
Weekly Epidemiological Record 1988 No.1-2 ~ No.52
Weekly Epidemiological Record 1989 No.1 ~ No.51-52
Weekly Epidemiological Record 1990 No.1 ~ No.51-52
Weekly Epidemiological Record 1991 No.1-2 ~ No.51-52
Weekly Epidemiological Record 1992 No.1-2 ~ No.51-52
Weekly Epidemiological Record 1993 No.1-2 ~ No.52
Weekly Epidemiological Record 1994 No.1 ~ No.51-52
Weekly Epidemiological Record 1995 No.1 ~ No.51-52
Weekly Epidemiological Record 1996 No.1 ~ No.51-52
Weekly Epidemiological Record 1997 No.1-2 ~ No.51-52
Weekly Epidemiological Record 1998 No.1-2 ~ No.51-52
Weekly Epidemiological Record 1999 No.1~ No.51-52

Weekly Epidemiological Record 2000 No.1 ~ No.51-52
Weekly Epidemiological Record 2001 No.1 ~ No.51-52
Weekly Epidemiological Record 2002 No.1 ~ No.51-52
Weekly Epidemiological Record 2003 No.1-2 ~ No.51-52
Weekly Epidemiological Record 2004 No.1-2 ~ No.51-52
Weekly Epidemiological Record 2005 No.1 ~ No.51-52
Weekly Epidemiological Record 2006 No.1 ~ No.51-52
Weekly Epidemiological Record 2007 No.1-2 ~ No.51-52
Weekly Epidemiological Record 2008 No.1 ~ No.51-52
Weekly Epidemiological Record 2009 No.1-2 ~ No.51-52
Weekly Epidemiological Record 2010 No.1-2 ~ No.51-52
Weekly Epidemiological Record 2011 No.1-2 ~ No.51-52
Weekly Epidemiological Record 2012 No.1 ~ No.51-52
Weekly Epidemiological Record 2013 No.1 ~ No.52

(3) Organization: WHO Collaborating Centre for Reference and Research on Influenza.

Reports: Reports and newsletters.

Access: http://www.influenzacentre.org/centre_reports.htm.

Detail:

Annual report 2006
Annual report 2010
Annual report 2011
Annual report 2012

(4) Organization: Australian Government Department of Health.

Reports: National Influenza Surveillance Scheme annual reports.

Access: http://www.health.gov.au/internet/main/publishing.nsf/Content/cda-pubs-annlrpt-fluannrep.htm.

Detail:

National influenza surveillance 1994 – annual report
National influenza surveillance 1995 – annual report
National influenza surveillance 1996 – annual report
National influenza surveillance 1997 – annual report
National influenza surveillance 1998 – annual report
National influenza surveillance 1999 – annual report
National influenza surveillance 2000 – annual report
National influenza surveillance 2001 – annual report
National influenza surveillance 2002 – annual report
National influenza surveillance 2003 – annual report
National influenza surveillance 2004 – annual report
National influenza surveillance 2005 – annual report
National influenza surveillance 2006 – annual report
National influenza surveillance 2007 – annual report
National influenza surveillance 2008 – annual report

(5) Organization: Public Health Surveillance from for New Zealand.

Access: https://surv.esr.cri.nz/PDF_surveillance/Virology/FluVac/
Detail:
Recommendation for the influenza vaccine composition 2005
Recommendation for the influenza vaccine composition 2006
Recommendation for the influenza vaccine composition 2007
Recommendation for the influenza vaccine composition 2008
Recommendation for the influenza vaccine composition 2009
Recommendation for the influenza vaccine composition 2010
Recommendation for the influenza vaccine composition 2011
Recommendation for the influenza vaccine composition 2012
Recommendation for the influenza vaccine composition 2013

(6) Organization: European Centre for Disease Prevention and Control.
Access:
http://www.ecdc.europa.eu/en/PUBLICATIONS/surveillance_reports/Pages/index.aspx
Detail:
Influenza virus characterization. Summary Europe, January 2010
Influenza virus characterization. Summary Europe, February 2010
Influenza virus characterization. Summary Europe, March 2010
Influenza virus characterization. Summary Europe, April 2010
Influenza virus characterization. Summary Europe, May 2010
Influenza virus characterization. Summary Europe, June 2010
Influenza virus characterization. Summary Europe, July 2010
Influenza virus characterization. Summary Europe, August 2010
Influenza virus characterization. Summary Europe, September 2010
Influenza virus characterization. Summary Europe, December 2010
Influenza virus characterization. Summary Europe, February 2011
Influenza virus characterization. Summary Europe, March 2011
Influenza virus characterization. Summary Europe, April 2011
Influenza virus characterization. Summary Europe, May-June 2011
Influenza virus characterization. Summary Europe, July 2011
Influenza virus characterization. Summary Europe, August and September 2011
Influenza virus characterization. Summary Europe, December 2011
Influenza virus characterization. Summary Europe, February 2012
Influenza virus characterization. Summary Europe, March 2012
Influenza virus characterization. Summary Europe, June 2012
Influenza virus characterization. Summary Europe, July 2012
Influenza virus characterization. Summary Europe, September 2012
Influenza virus characterization. Summary Europe, October 2012
Influenza virus characterization. Summary Europe, November 2012
Influenza virus characterization. Summary Europe, December 2012
Influenza virus characterization. Summary Europe, February 2013
Influenza virus characterization. Summary Europe, March 2013
Influenza virus characterization. Summary Europe, April 2013
Influenza virus characterization. Summary Europe, May 2013

Influenza virus characterization. Summary Europe, June 2013
Influenza virus characterization. Summary Europe, July 2013
Influenza virus characterization. Summary Europe, September 2013

(7) Organization: National Institute for Medical Research.

Access: http://www.nimr.mrc.ac.uk/who-influenza-centre/annual-and-interim-reports/

Detail:

Annual report 2002
Annual report 2003
Annual report 2004
Interim Report February 2005
Interim Report September 2005
Interim Report March 2006
Interim Report September 2006
Interim Report March 2007
Interim Report September 2007
Interim Report March 2008
Interim Report September 2008
Interim Report February 2009
Interim Report September 2009
Interim Report February 2010
Interim Report September 2010
Interim Report February 2011
Interim Report September 2011
Interim Report February 2012
Interim Report September 2012
Interim Report February 2013
Interim Report September 2013

(8) Published papers

Baek, Y.H., et al. (2009) Molecular characterization and phylogenetic analysis of H3N2 human influenza A viruses in Cheongju, South Korea, Journal of microbiology, 47, 91-100.

Pechirra, P., et al. (2008) Characterization of influenza A/Fujian/411/2002(H3N2)-like viruses isolated in Portugal between 2003 and 2005, Journal of medical virology, 80, 1624-1630.

de Jong, J.C., et al. (2007) Antigenic and genetic evolution of swine influenza A (H3N2) viruses in Europe, Journal of virology, 81, 4315-4322.

Iorio, A.M., et al. (2006) An influenza A/H3 outbreak during the 2004/2005 winter in elderly vaccinated people living in a nursing home, Vaccine, 24, 6615-6619.

Daum, L.T., et al. (2005) Influenza A (H3N2) outbreak, Nepal, Emerging infectious diseases, 11, 1186-1191.

Campitelli, L., *et al.* (2002) H3N2 influenza viruses from domestic chickens in Italy: an increasing role for chickens in the ecology of influenza?, *The Journal of general virology*, 83, 413-420.

Hay, A.J., et al. (2001) The evolution of human influenza viruses, Philosophical transactions of the Royal Society of London. Series B, Biological sciences, 356, 1861-1870.

Coiras, M.T., et al. (2001) Rapid molecular analysis of the haemagglutinin gene of human influenza A

H3N2 viruses isolated in spain from 1996 to 2000, Archives of virology, 146, 2133-2147.
Ellis, J.S., Chakraverty, P. and Clewley, J.P. (1995) Genetic and antigenic variation in the haemagglutinin of recently circulating human influenza A (H3N2) viruses in the United Kingdom, *Archives of virology*, 140, 1889-1904.
Castrucci, M.R., et al. (1994) Antigenic and sequence analysis of H3 influenza virus haemagglutinins from pigs in Italy, The Journal of general virology, 75 (Pt 2), 371-379.
Nakajima, S., Takeuchi, Y. and Nakajima, K. (1988) Location on the evolutionary tree of influenza H3 haemagglutinin genes of Japanese strains isolated during 1985-6 season, Epidemiology and infection, 100, 301-310.
Both, G.W., et al. (1983) Antigenic drift in influenza virus H3 hemagglutinin from 1968 to 1980: multiple evolutionary pathways and sequential amino acid changes at key antigenic sites, Journal of virology, 48, 52-60.

Supplementary Note 3. Preprocessing of antigenic distance parameter

The antigenic distance between two strains a and b was defined as following equation:

$$D_{ab} = log\sqrt{\frac{H_{aa}H_{bb}}{H_{ab}H_{ba}}} \qquad (1)$$

The HI titer $H_{ab}$is the maximum dilution of serum raised again strain a, which is necessary to inhibit cell agglutination caused by strain b. Two viruses were defined as antigenic variants when the $log^{-1}D_{ab}$ was above 4, otherwise, the pair was treated as antigenic similar. Due to the different experimental conditions, for the same strain pair, the HI measures collected from different reports existed difference. To avoid this affection, for the HI values of same strain pair derived from different reports, the outlines defined as those with $|D_{ab} - \overline{D_{ab}}|$ ranked within top 10% in descending order were abandoned. After that, if all classified as antigenic similar or antigenic difference, those strain pairs will remain and be labeled. Finally, the intersection of the HI assays and the sequence set were generated as our dataset. This set contains 3867 pairs involving 288 HA proteins with 2286 antigenic variants and 1581 antigenic similar. Data ranging from 2011 to 2013 were selected as the independent dataset to evaluate the performance of this model.

Supplementary Note 4. Model parameterization, Definition of antigenic similar

Different parameters of “spin-image” plane and seed distance were tested to evaluate the model robustness, as well as to balance accuracy and computational complexity. As at least 95% of the epitope residues can be contained by a 20Å * 40Å (-30 Å to 10 Å) plane (**Supplementary Fig. 7**), it was chose as the size of our spin-image in this study.

For inter- pathogen case of influenza HA protein, the optimized resolution of spin-image was defined as 0.5 (horizontal) * 0.5 (vertical), the cutoff of $SS_{AB} = 0.9$ was derived by adopt the optimal point in ROC curve. Further, for better understanding, SS_{AB} was translated to theoretical antigenicity distance (TD_{ab}) with cutoff set as 4 (**Supplementary Note 3**). For intra- pathogen case of E protein of DENV and ZIKV, the optimized resolution of spin-image was defined as 2 (horizontal) * 2 (vertical), the cutoff of SS_{AB} was set as 0.7.

Supplementary Note 5. Experimental validation of influenza H3

Design of Potential Broad-Spectrum Vaccine

By aligning all HA1 sequences of influenza A/H3N2 from year 2006 to 2009, a consensus sequence ("Con H3") was designed by taking the dominant residues at each position. The "Con H3" nucleotide sequences were chemically synthesized by Shanghai Generay Biotech Co, Ltd and then constructed onto the DNA vaccine vector pVKD1.0 from VacDiagn Biotechnology (Suzhou, China), resulting the DNA vaccine pVKD1.0-CON H3. Then DNA vaccines were prepared by endo-free plasmid Giga kit (QIAGEN) for subsequent animal immunization.

Animal's vaccination and Isolation of mAbs

All animal experiments were performed in accordance with the guidelines of Soochow University Animal Care and Use Committee. Animals were allowed access to food and water ad libitum and kept on a 12 hour light/dark cycle. Specific pathogen free (SPF) female 6-8 week old BALB/c mice (Shanghai Slaccas, China) were administered intramuscularly (i.m.) DNA vaccines three times at the interval of two weeks. Two weeks after the final inoculation of vaccines, mice were sacrificed and serum were harvested and used in the subsequent neutralization assay.

Meanwhile, five additional SPF female 6-8 week old BALB/c mice (Shanghai Slaccas, China) were administrated above vaccine regimen. Two weeks after the last immunization, mice were sacrificed and splenocytes were collected to fuse with SP2/0-Ag14 myeloma cell lines. Hybridomas were screened with the pseudovirus based neutralization assays. Antibodies were purified by HiTrap Protein G HP columns (GE Healthcare, NJ) from supernatants of positive hybridomas.

Pseudovirus preparation

All pseudotyped viruses were provided by VacDiagn Biotechnology (Suzhou, China). Briefly, plasmids pVKD1.0-HA expressing HAs derived from different influenza strains (**Supplementary Table 1**) and plasmids pVKD1.0-NA expressing corresponding NA derived from the identical influenza strain, and lentivirus backbone plasmid pNL4.3-luc E.R. were co-transfected with TurboFect transfection reagent (Thermo Scientific, USA) in 293T package cells. After 48 hours of incubation, the supernatant containing pseudoviruses was collected, filtered and stored as working solution. The genomic RNA of the pseudovirus carries luciferase reporter gene, thus its infectivity is quantified by luciferase activity in virus infected cells. All pseudoviruses were titrated in Madin-Darby canine kidney (MDCK) cells and calculated the tissue culture infectious dose (TCID50).

Neutralization assay

3-fold serially diluted serum or mAb sample were incubated with 200 TCID50 pseudoviruses at the final volume of 150 μL at 37°C for 1 hour, then the mixture was added to culture of MDCK cells. After 4 hours incubation, cells were washed with phosphate buffered saline (PBS) and cultured in complete DMEM media for 48 hours. Infected cells were lysed by luciferase lysis buffer (Promega, Madison, WI) and relative luminescence units (RLU) were measured by luciferase substrate (Promega, Madison, WI). Inhibitory percent was calculated as: (average RLU of virus control wells - RLU of sample at a given dilution) / average RLU of virus control wells. The inhibitory curve was plotted and the 50% inhibitory concentration (IC50) was calculated by Graphpad Prism.

Supplementary References

1 Liao, Y. C., Ko, C. Y., Tsai, M. H., Lee, M. S. & Hsiung, C. A. ATIVS: analytical tool for influenza virus surveillance. *Nucleic acids research* **37**, W643-646, doi:10.1093/nar/gkp321 (2009).

2 Smith, D. J. *et al.* Mapping the antigenic and genetic evolution of influenza virus. *Science* **305**, 371-376, doi:10.1126/science.1097211 (2004).

3 Wiley, D. C., Wilson, I. A. & Skehel, J. J. Structural identification of the antibody-binding sites of Hong Kong influenza haemagglutinin and their involvement in antigenic variation. *Nature* **289**, 373-378 (1981).

4 Qiu, J. X., Qiu, T. Y., Yang, Y. Y., Wu, D. F. & Cao, Z. W. Incorporating structure context of HA protein to improve antigenicity calculation for influenza virus A/H3N2. *Scientific reports* **6**, doi:Artn 31156
10.1038/Srep31156 (2016).

5 Lees, W. D., Moss, D. S. & Shepherd, A. J. A computational analysis of the antigenic properties of haemagglutinin in influenza A H3N2. *Bioinformatics* **26**, 1403-1408, doi:10.1093/bioinformatics/btq160 (2010).

6 Yang, J. L., Zhang, T. & Wan, X. F. Sequence-Based Antigenic Change Prediction by a Sparse Learning Method Incorporating Co-Evolutionary Information. *PloS one* **9**, doi:ARTN e106660
10.1371/journal.pone.0106660 (2014).

7 Shatsky, M., Nussinov, R. & Wolfson, H. J. A method for simultaneous alignment of multiple protein structures. *Proteins* **56**, 143-156, doi:10.1002/prot.10628 (2004).

8 Menke, M., Berger, B. & Cowen, L. Matt: local flexibility aids protein multiple structure alignment. *Plos Comput Biol* **4**, e10, doi:10.1371/journal.pcbi.0040010 (2008).

9 Zhang, Y. & Skolnick, J. TM-align: a protein structure alignment algorithm based on the TM-score. *Nucleic acids research* **33**, 2302-2309, doi:10.1093/nar/gki524 (2005).

10 Yang, Y., Zhan, J., Zhao, H. & Zhou, Y. A new size-independent score for pairwise protein structure alignment and its application to structure classification and nucleic-acid binding prediction. *Proteins* **80**, 2080-2088, doi:10.1002/prot.24100 (2012).

11 Powers, D. M. W. Evaluation: From Precision, Recall and F-Measure to ROC, Informedness, Markedness & Correlation. *Journal of Machine Learning Technologies* **2** 37-63 (2011).
