# Supplementary material for: CE-BLAST makes it possible to compute antigenic similarity for newly emerging pathogens
Source: Nat Commun. 2018 May 2;9:1772. doi: 10.1038/s41467-018-04171-2 (PMC5932059; doi:10.1038/s41467-018-04171-2)
Supplement: Supplementary file 3 — Description of Additional Supplementary Files [file 41467_2018_4171_MOESM3_ESM.pdf]

Description of Additional Supplementary Files

File Name: Supplementary Data 1
Description: **Normalized antisera data for dengue virus**

File Name: Supplementary Data 2
Description: **HI-test data for influenza virus A/H3N2**

File Name: Supplementary Data 3
Description: **Sequence data for influenza virus A/H3N2**
